# Theoretical spectroscopic study of acetyl (CH _3_CO), vinoxy (CH _2_CHO), and 1-methylvinoxy (CH _3_COCH _2_) radicals. Barrierless formation processes of acetone in the gas phase

**DOI:** 10.12688/openreseurope.14073.1

**Published:** 2021-09-30

**Authors:** Hamza El Hadki, Victoria Guadalupe Gámez, Samira Dalbouha, Khadija Marakchi, Oum Keltoum Kabbaj, Najia Komiha, Miguel Carvajal, Maria Luisa Senent Diez

**Affiliations:** 1 Laboratoire de Spectroscopie, Modélisation Moléculaire, Matériaux, Nanomatériaux, Eau et Environnement, LS3MN2E/CERNE2D, Faculté des Sciences Rabat, Université Mohammed V, Rabat, BP1014, Morocco; 2 Departamento de Química y Física Teóricas, IEM-CSIC, Unidad Asociada GIFMAN, CSIC-UHU, Madrid, 28006, Spain; 3 Equipe de recherche : Matériaux et Applications Environnementales, Laboratoire de Chimie Appliquée et Environnement, Département de chimie, Faculté des Sciences d’Agadir, Université Ibn Zohr, Agadir, B.P 8106, Morocco; 4 Departamento de Ciencias Integradas, Centro de Estudios Avanzados en Física, Matemática y Computación; Unidad Asociada GIFMAN, CSIC-UHU, Universidad de Huelva, Huelva, 21071, Spain; 5 Instituto Universitario Carlos I de Física Teórica y Computacional, University of Granada, Granada, Spain

**Keywords:** acetone, CH3CO, CH2CHO, CH3COCH2, radical, spectrum, barrierless, LAM

## Abstract

**Background: **Acetone is present in the earth´s atmosphere and extra-terrestrially. The knowledge of its chemical history in these environments represents a challenge with important implications for global tropospheric chemistry and astrochemistry. The results of a search for efficient barrierless pathways producing acetone from radicals in the gas phase are described in this paper. The spectroscopic properties of radicals needed for their experimental detection are provided.

**Methods: **The reactants were acetone fragments of low stability and small species containing C, O and H atoms. Two exergonic bimolecular addition reactions involving the radicals CH _3_, CH _3_CO, and CH _3_COCH _2_, were found to be competitive according to the kinetic rates calculated at different temperatures. An extensive spectroscopic study of the radicals CH _3_COCH _2_ and CH _3_CO, as well as the CH _2_CHO isomer, was performed. Rovibrational parameters, anharmonic vibrational transitions, and excitations to the low-lying excited states are provided. For this purpose, RCCSD(T)-F12 and MRCI/CASSCF calculations were performed. In addition, since all the species presented non-rigid properties, a variational procedure of reduced dimensionality was employed to explore the far infrared region.

**Results: **The internal rotation barriers were determined to be V _3_=143.7 cm ^-1^ (CH _3_CO), V _2_=3838.7 cm ^-1^ (CH _2_CHO) and V _3_=161.4 cm ^-1^ and V _2_=2727.5 cm ^-1^ (CH _3_COCH _2_).The splitting of the ground vibrational state due to the torsional barrier have been computed to be 2.997 cm ^-1^, 0.0 cm ^-1^, and 0.320 cm ^-1^, for CH _3_CO, CH _2_CHO, and CH _3_COCH _2_, respectively.

**Conclusions: **Two addition reactions, H+CH _3_COCH _2_ and CH _3_+CH _3_CO, could be considered barrierless formation processes of acetone after considering all the possible formation routes, starting from 58 selected reactants, which are fragments of the molecule. The spectroscopic study of the radicals involved in the formation processes present non-rigidity. The interconversion of their equilibrium geometries has important spectroscopic effects on CH _3_CO and CH _3_COCH _2_, but is negligible for CH _2_CHO.

## Plain language summary

In addition to its industrial applications, acetone (CH _3_COCH _3_), the smallest ketone, is present in gas phase environments such as the earth´s atmosphere and extraterrestrial sources. The knowledge of its chemical history in these environments, as well as that of other volatile molecular species, represents a challenge with important implications for global tropospheric chemistry. Organic radicals can play important roles in the chemical evolution.

In this paper gas phase barrierless processes and the corresponding properties were studied at different temperatures. Probable exergonic bimolecular addition processes, which reactants are acetone fragments or small neutral species observed in the gas phase sources containing C, O and H atoms, were described. In addition, this paper is devoted to the theoretical spectroscopic study of three radicals: the acetyl radical (CH _3_CO), the vinoxy radical (CH _2_CHO), and the 1-methylvinoxy radical (CH _3_COCH _2_). The three systems were acetone fragments and potential reactants. Our main objective is to provide the theoretical point of view to help further spectroscopic studies of these not-yet-fully characterized species that can play important roles in the gas phase chemistry.

## Introduction

In addition to its industrial applications, acetone (CH _3_COCH _3_), the smallest ketone, is present in gas phase environments such as the earth´s atmosphere and the interstellar medium ^1– 5^. The knowledge of its chemical history in these environments, as well as that of other volatile molecular species, represents a challenge with important implications for global tropospheric chemistry and astrochemistry. Organic radicals can play important roles in the chemical evolution of the terrestrial atmosphere and extra-terrestrial gas phase sources.

Atmospheric acetone arises from both natural and anthropogenic sources ^1, 2^. It is naturally produced by vegetation that emits large quantities of nonmethane organic compounds. In the troposphere, these biogenic compounds can undergo photolysis and react with OH and NO _3_ radicals, and ozone, resulting in the formation of oxygenated products such as ketones ^2, 5, 6^. On the other side, acetone is a major source of hydrogen oxide radicals (HO _x_) and peroxyacetyl nitrate through photolysis ^2, 6^. Decomposition of acetone can occur in the presence of OH to produce radicals such as CH _3_COCH _2_^7^. The OH-initiated oxidation of acetone in the presence of NO in air has been shown to form the acetonoxy radical [CH _3_COCH _2_O], which undergoes rapid decomposition under atmospheric conditions to form HCHO and CH _3_CO ^1^.

Acetone was the first ten-atom molecule to be detected in the interstellar medium ^4, 5^. Recently, it has been observed in the gas phase in various extraterrestrial environments ^8, 9^. It is generally accepted that organic compounds are formed in the interstellar medium on ice mantles, although the possibility of gas phase processes is not ignored ^10^. At the temperatures and pressures of the interstellar medium, barrierless processes can be competitive with respect to those involving ice mantles ^11^. Barrierless processes involve low stability species such as radicals, charged or unsaturated molecules ^11^.

 Following the same methodology employed in a previous work ^11^, for this paper, gas phase barrierless processes and the corresponding kinetic rates were studied at different temperatures. Probable exergonic bimolecular addition processes, the reactants which are acetone fragments or small neutral species observed in the gas phase sources containing C, O and H atoms, are described. In addition, this paper is devoted to the theoretical spectroscopic study of three radicals: the acetyl radical (CH _3_CO), the vinoxy radical (CH _2_CHO), and the 1-methylvinoxy radical (CH _3_COCH _2_). The three systems were acetone fragments and potential reactants. Our main objective is to provide perspective from the *ab initio* calculations and to help further spectroscopic studies of these three, not yet fully characterized species that can play important roles in the gas phase chemistry involving organic molecules, such as diacetyl or pyruvic acid ^12, 13^.

 The rotational spectra of CH _3_CO and CH _2_CHO were measured by Hirota *et al.* ^14^, Endo *et al.* and Hansen *et al.* ^15, 16^, respectively. As far we know, experimental rotational parameters are not available for CH _3_COCH _2_.

 The vibrational spectra of the CH _3_CO, CH _2_CHO, and CH _3_COCH _2 _radicals were studied in Ar matrices by Jacox ^17^, Shirk *et al.* ^18^, and Lin *et al.* ^19^, respectively, whereas Das and Lee ^20^ addressed the infrared spectrum of CH _3_CO in solid p-H _2_. In the gas phase, previous measurements corresponding to CH _2_CHO ^21– 27^ and CH _3_COCH _2_^28^ are accessible, whereas for CH _3_CO experimental vibrational data in the gas phase are unavailable.

Low-lying electronic transitions were previously measured for the three radicals. The CH _3_CO radical photodecomposes into CH _3_+CO in the visible region ^17^. The ground electronic state presented a doublet multiplicity character. In addition, three excited electronic doublet states have been explored ^29– 32^. Band centers were determined to lie at 2.3 eV ^29^ and in the 200-240 nm (5.1-6.2 eV) region ^30^. Maricq *et al.* ^31^ observed bands at 217 nm (2.3 eV) and in the 240-280 nm (4.4-5.1 eV) region, which were confirmed by Cameron *et al.* ^32^. For the CH _2_CHO isomer, the A(A”)←X(A”) and B(A”)←X(A”) transitions has been centered at 1.0 eV ^26, 33, 34^ and at 3.6 eV ^22– 25, 33, 35, 36^ using different experimental techniques. The B(A”)←X(A”) transition of CH _3_COCH _2 _was found at 3.4 eV by Williams *et al.* ^37, 38^ who also provided internal rotation barriers. To our knowledge, all the previous experimental data concerned doublet states. No information is available concerning quartet states. Since one of our objectives was to localize the excited states, all the previous data is summarized with our results.

 Theoretical techniques have also been applied to the study of the three radicals ^39– 41^. Mao *et al.* ^39^ have determined vertical excitation energies to four excited doublet states of CH _3_CO using multireference single and double excitation configuration interaction. The work of Yamaguchi *et al.* ^40^ focused on the CH _2_CHO, and CH _3_COCH _2_ radicals.

 For this new paper, we tackle to different spectroscopic properties with special attention to the far infrared region, relevant for the interpretation of rovibrational spectra. This new paper is organized as follows: The “computational tools” section under the Methods contains information about the electronic structure computations and computer codes. The next section presents the results and the discussion about the barrierless formation processes of acetone and the corresponding kinetic rates; the spectroscopic characterization of the radicals using two different procedures (one of them suitable for species with large amplitude vibrations and the far infrared region), is explored. Our recent studies of acetone ^42^ and the CH _3_OCH _2_ radical ^43^ are examples of this last procedure. Finally, the conclusions are drawn.

## Methods

### Computational tools

Different levels of electronic structure theory were combined taking into consideration the computational requirements of the reactive processes and the spectroscopic studies.

***Formation processes.*** The search for the possible barrierless formation processes was performed using the Searching Tool for Astrochemical Reactions ( STAR) online tool ^44^. This statistical tool contains a broad and comprehensive molecular database. Implemented algorithms search for all the likely combinations of reactants leading to a specific molecular species. The processes producing the desired molecule and some subproducts are automatically rejected if the viability of these last species cannot be confirmed according to the Gibbs free energy. The viability is considered confirmed when species are listed in the data base. The corresponding minimum energy paths were computed using the density functional theory (DFT) ^45^ and the 6-311+G(d,p) basis set ^46^ as it is implemented in the GAUSSIAN 16 software ^47^. The thermochemical properties were determined by optimizing the geometry with the CBS-QB3 procedure ^48^, a complete basis set model implemented in GAUSSIAN modified for the use of the B3LYP hybrid density functional. The corresponding kinetic rate constants were computed using POLYRATE version 2017 software ^49^.

***Spectroscopic and geometrical properties.*** The ground electronic state structure of the three radicals and the corresponding harmonic frequencies were computed using explicitly correlated coupled cluster theory with single and double substitutions, augmented by a perturbative treatment of triple excitations, RCCSD(T)-F12 ^50, 51^ implemented in MOLPRO version 2012.1 ^52^. The default options and a basis set denoted by AVTZ-F12, were employed. AVTZ-F12 contains the aug-cc-pVTZ (AVTZ) ^53^ atomic orbitals, the corresponding functions for the density fitting, and the resolutions of the identity. The core-valence electron correlation effects on the rotational constants were introduced using RCCSD(T) ^54^ and the cc-pCVTZ basis set (CVTZ) ^55^.

The three radicals can be defined as nonrigid molecules because they show large amplitude motions that interconvert different minima of the potential energy surface (PES). Then, two different theoretical models were combined to determine the spectroscopic parameters, vibrational second order perturbation theory (VPT2) ^56^ implemented in GAUSSIAN, and a variational procedure of reduced dimensionality which is detailed in our papers ^57– 59^.

If VPT2 is applied, a unique minimum is postulated to exist in the potential energy surfaces, the radicals are assumed to be semi-rigid and all the vibrations are described as small displacements around the equilibrium geometry. For CH _3_CO, CH _2_CHO, and CH _3_COCH _2_, anharmonic spectroscopic frequencies were obtained from anharmonic force fields, computed using second order Møller-Plesset theory (MP2) and the VQZ, VQZ, and AVTZ basis sets ^52^, respectively.

If the variational procedure is applied, the non-rigidity is taken into account and the minimum interconversion is considered implicitly. For this purpose, RCCSD(T)-F12/AVTZ potential energy surfaces were computed, and later on they were vibrationally corrected at the MP2/VQZ, MP2/VQZ, and MP2/AVTZ level of theory, respectively. 

Vertical excitation energies to the excited electronic states were computed using MRCI/CASSCF theory ^60, 61^. For the two small radicals, the active space was constructed with eight a’ and four a” orbitals and 13 electrons. The five a’ internal orbitals, doubly occupied in all the configurations, were optimized. In the case of CH _3_COCH _2_, the active space was built using nine a’ and four a” orbitals and fifteen electrons, whereas eight a’ orbitals were optimized but they were doubly occupied in all the configurations.

## Results and discussion

### Barrierless formation processes of acetone: the function of radicals

In the gas phase, efficient reactions for competing with formation processes on ice surfaces are those which follow barrierless pathways and involve low stability species. To establish some limits to the present work, we choose the set of reactants shown in Table 1. In principle, they obey the following conditions: (1) they enclose the atomic elements H, C, and O constituents of acetone; (2) they contain at most eleven atoms, as the objective is to select chemical routes of increasing molecular complexity; (3) they have been detected in the gas phase of the ISM or, at least, they are listed as probably detectable species. We detail this procedure in our previous paper on the C _3_O _3_H _6_ isomers ^11^.

**Table 1.** List of selected reactants (N _a_ = number of atoms).

*N _a_*	*Reactants*
1	^•^H
2	H _2_; ^3^O _2_; C _2_; OH ^•^; CH ^•^; OH ^+^; CH ^+^; CO ^•+^; CO
3	C _3_; HOC ^+^; C _2_H ^•^; H _2_O; C _2_O; H _2_O ^•+^; ^3^CH _2_; H _3_^+^; CO _2_; HCO ^+^; HCO ^•^
4	HOCO ^+^; l-C _3_H ^•^; CH _3_^•^; H _2_CO; HCOH; C _3_O; HCOO ^•^; C _2_H _2_; HOCO ^•^; H _3_O ^+^; HCCO
5	^•^CH _2_OH; C _4_H ^•^; CH _3_O ^•^; C _4_H ^-^; HCOOH; CH _4_; l-C _3_H _2_; CH _2_CO
6	C _2_H _4_; CH _3_CO ^•^; CH _3_OH; ^•^CH _2_CHO; HC _2_CHO; H _2_C _4_; HC _4_H
7	CH _3_CHO; CH _2_CHOH; CH _3_C _2_H
8	CH _2_CHCHO; CH _3_OCH _2_^•^; CH _3_CHOH
9	CH _3_C _4_H; CH _3_CH _2_OH; CH _3_COCH _2_^•^; CH _3_CCH _3_; CH _3_CHCH _2_

 All the possible chemical routes starting from the selected reactants were automatically generated by the STAR software ^44^. This statistical tool contains a broad and comprehensive molecular database. Implemented algorithms search for all likely combinations of reactants leading to a specific molecular species. The processes producing the desired molecule and some by-products are automatically rejected if the viability of these last species cannot be confirmed. The tool selects the exergonic processes for which the exchange of Gibbs free energies is negative (∆G < 0). The selected processes must occur following a limited number of steps. The number of steps is considered to concur with the sum of breaking and forming bonds. To quantify them, the maximum number of necessary elementary steps (MNES) parameter is defined.

In principle, 75 exergonic processes are derived from STAR. For all of them, ∆G was computed by optimizing the geometry using the CBS-QB3 procedure ^48^. Fourteen final exergonic reactions for which MNES < 3 were found, but only two of them could be considered as barrierless processes (see Table 2). Energy profiles proving these findings were computed at the M05-2X/6-311+G(d,p) level of theory ^45, 46^. They represent minimum energy paths.

**Table 2.** Rate constants (cm ^3^molecule ^-1^ s ^-1^).

	*Reaction*	200K		298K	500K	1000K
		CVT	µVT	CVT	CVT	CVT
1	H ^•^ + CH _3_COCH _2_→ CH _3_COCH _3_	6.3E-21	0.0E+00	2.6E-17	8.2E-15	5.2E-13
2	CH _3_^•^ + CH _3_CO ^•^ → CH _3_COCH _3_	6.0E-21	0.0E+00	2.4E-17	2.3E-14	3.3E-12

 The kinetic rate constants of the processes are summarized in Table 2. They were evaluated using the single-faceted variable-reaction-coordinate variational transition state theory (VRC-VTST) ^62, 63^ implemented in POLYRATE ^49^. The rate constants obey the following equation:


k(T,s)=ℏ22πgeσ1σ2σ≠Q1Q2(2πμkBT)32∫dEe−EKBTdJN(E,J,s)(1)

In this equation, s and T denote the reaction coordinate and the temperature, respectively; *g _e_* represents the rate between the electronic partition function of the transition state and the product of the electronic partition function of the two reactants; μ, Q _1_ and Q _2_ designate the reduced mass and the rotational partition functions of the reactants. J is the angular momentum quantum number and N (E, J, s) represents the number of allowed states corresponding to the E energy. σ _1_, σ _2_, and σ ^≠^ denote the cross-sections for the two reactants and for the transition states.

 The starting points of the rate computation were the M05-2X/6-311+G(d,p) energies, geometries and harmonic fundamentals of reactants and products computed along the pathways. The s reaction coordinate was allowed to vary from 1.8 to 4.2 Å with intervals of 0.2 Å. The rates were computed at fifteen different T (200, 210, 220, 230, 240, 250, 260, 280, 298, 300, 400, 500, 700, 900 and 1000K). The number of allowed states was determined in a Monte Carlo simulation considering all the possible orientations. The rates at 200K (middle point of the interstellar Hot Core temperature range, 100-300K), 298K (room temperature), and 500K, corresponding to the 29 barrierless processes, are shown in Table 2. Rates were computed using the canonical variational transition state theory (CVT), which is the conventional theory. However, as CVT is not recommended for very low temperatures, rates at 200K were also computed using microcanonical variational transition state theory (μVT) with multidimensional semiclassical approximations for tunneling and nonclassical reflection ^64, 65^.

### Spectroscopic characterization of CH _3_CO, CH _2_CHO, and CH _3_COCH _2_

***Ground electronic state: equilibrium structures.*** The study of the rovibrational properties of acetone and its monosubstituted isotopologues using *ab initio* calculations was the aim of a previous work from some of the authors of the present paper ^42^. In this new paper, we attend, using a similar methodology, the structural and spectroscopic parameters of the three radicals, CH _3_CO, CH _2_CHO, and CH _3_COCH _2_, involved in acetone gas phase processes.

**Figure 1.**
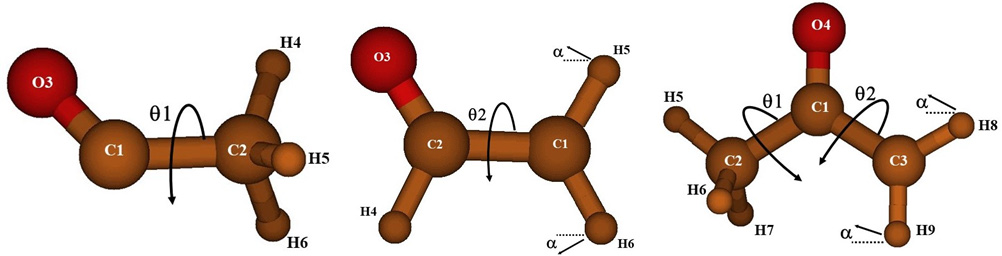
The preferred geometries of the CH _3_CO, CH _2_CHO, and CH _3_COCH _2_ radicals.

Table 3 collects the RCCSD(T)-F12/AVTZ structural parameters and the equilibrium rotational constants of the preferred geometries of the three radicals in their ground electronic state. The three geometries can be classified in the C _s_ point group. The MRCI/CASSCF/AVTZ dipole moment components are also shown. Figure 1 represents those preferred geometries. 

**Figure 2.**
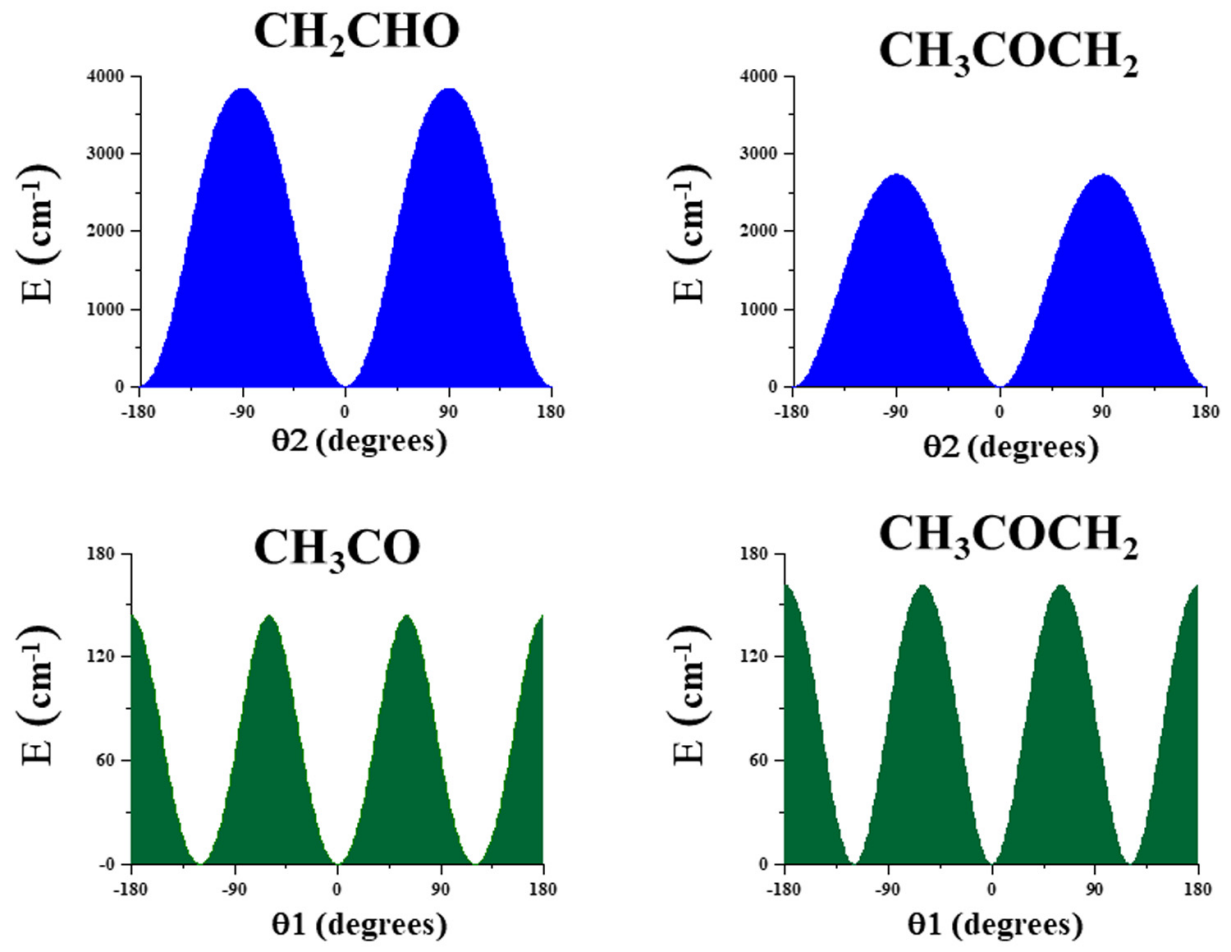
Internal rotation barriers.

The three radicals display internal rotation that interconvert equivalent minima separated by barriers. θ _1_ and θ _2_ denote the CH _3_ and the CH _2_ torsional coordinates, respectively. Using RCCSD(T) theory, the energy barriers were computed to be V _3_=143.7 cm ^-1^ (CH _3_CO), V _2_=3838.7 cm ^-1^(CH _2_CHO), and V _3_=161.4 cm ^-1^ and V _2_=2727.5 cm ^-1^ (CH _3_COCH _2_). A third coordinate, α (the CH _2_ wagging) interacts strongly with the CH _2_ torsion in two of the three species. Figure 2 represents one-dimensional cuts of the ground electronic state potential energy surfaces that emphasize the torsional barriers.

**Table 3.** RCCSD(T)-F12/AVTZ relative energies (E, E ^ZPVE^, in cm ^-1^), internal rotation barriers (V _3_, V _2_, in cm ^-1^). Rotational constants (in MHZ), MRCI/CASSCF/AVTZ dipole moment (in D) and equilibrium structural parameters ( *distances, in Å, angles, in degrees)* of acetyl, vinoxy, and 1-methylvinoxy radicals.

	CH _3_CO ^a^ (X ^2^A’)	CH _2_CHO (X ^2^A”)	CH _3_COCH _2_^b^ (X ^2^A”)
**E**	0.0	2514.2	-
**E ^ZPVE^**	0.0	2498.8	-
**V _2_**	**-**	3838.7	2727.5
**V _3_**	143.7	-	161.4
**A _e_**	84134.35	67084.88	10934.76
**B _e_**	9982.57	11456.25	9115.74
**C _e_**	9444.29	9785.20	5128.93
**μ _a_**	0.2423	0.9465	0.5444
**μ _b_**	2.3244	2.6175	1.3272
**μ _c_**	0.0	0.0	0.0
**μ**	2.3281	2.7834	1.4346
**CH _3_CO**		**CH _2_CHO**	
C1C2	1.5116	C1C2	1.4325
O3C1	1.1816	O3C2	1.2290
H4C2	1.0912	H4C2	1.1014
H5C2=H6C2	1.0892	H5C1=H6C1	1.0809
O3C1C2	128.3	O3C2C1	122.8
H4C2C1	110.7	H4C2C1	117.0
H5C2C1=H6C2C1	108.4	H5C1C2	119.0
H4C2C1O3	0.0	H6C1C2	120.9
H5C2C1H4=-H6C2C1H4	121.6		
**CH _3_COCH _2_**			
C1C2	1.5129	C2C1O4	121.6
C1C3	1.4467	H5C2C1	109.5
O4C1	1.2291	H6C2C1=H7C2C1	110.2
H5C2	1.0869	C1C3H8	118.1
H6C2=H7C2	1.0917	H9C3H8	119.9
H8C3	1.0803	H5C2C1C3	180.0
H9C3	1.0814	H6C2C1H5= -H7C2C1H5	120.8
C2C1C3	117.9		
a) E=-152.989163 a.u.; b) E=-192.240093 a.u.			

It could be concluded that in CH _3_CO and CH _3_COCH _2_, the methyl torsional barrier is very low (V _3_ <200 cm ^-1^). A complex distribution of the torsional energy levels can be expected. With respect to the CH _2_ torsion, the barrier in CH _3_COCH _2_ is ~0.70 V _2_^CH2-CHO^ and of the same order of magnitude as in CH _3_OCH _2_^43^.

***Excited electronic states.*** Previous theoretical and experimental works resolved doublet multiplicity character of the electronic ground states. Nevertheless, using MRCI/CASSCF/AVTZ theory, we have identified the lowest excited electronic states to assure a clean enough ground electronic state to apply the used rovibrational models. Results are shown in Table 4 and were compared with previous experimental data ^22– 26, 29, 31, 33– 38^ or data from theoretical works ^39, 40^.

**Table 4.** Vertical excitation energies to the low-lying electronic states (in eV) computed with MRCI/CASSCF/AVTZ.

CH _3_CO ^a^			CH _2_CHO ^b^			CH _3_COCH _2_^c^		
	Calc.	Previous works		Calc.	Previous works		Calc.	
X ^2^A’	0.0	-	X ^2^A”	0.0		X ^2^A”	0.0	
A ^2^A”	2.4	2.33 ^29^ 2.6 ^39^	A ^2^A’	1.4	1.0 ^26, 33, 34^	A ^2^A’	2.2	
B ^2^A’	6.2	5.8 ^30^ (4.4-5.2) ^31^ 4.9 ^39^	B ^2^A”	4.4	3.57 ^22– 25, 33, 35, 36^ 3.4 ^40^	a ^4^A’	5.0	
a ^4^A”	6.3		a ^4^A’	4.9		B ^2^A”	5.7	3.4 ^37, 38^ 3.5 ^40^
b ^4^A’	6.7		C ^2^A’	5.4		b ^4^A”	6.4	
C ^2^A”	7.0	6.7 ^39^	b ^4^A”	7.2		C ^2^A’	6.8	

a) 29 Visible Absorption Spectrum; 30; Flash photolysis and kinetic spectroscopy; 31 Ultraviolet spectrum ; 39 MR(SD)CI calculations; b) 22– 25, 36 Laser induced fluorescence; 26, 34 Photoelectron spectroscopy; 33 Photochemical modulation spectroscopy; 35 Photodissociation spectroscopy; 40 MRCISD+Q/cc-pVDZ c) 37, 38 laser induced fluorescence; 40 MRCISD+Q/cc-pVDZ

The first excited electronic state of CH _3_CO and CH _3_COCH _2_ is a doublet state lying around 2 eV above the ground state. Vibronic effects are expected in the spectral region of the ground electronic state studied in this work. In the case of the CH _3_CO radical, the computed value is in good agreement with experiments ^29^. For CH _2_CHO, the vertical excitation to the first excited state was computed to be 1.4 eV in agreement with the experimental value of 1.0 eV ^26, 33, 34^. As a consequence, the first excited state can perturb the region of the H-stretching overtones. For the three species, the following higher excited states (doublets and quartets) lie over 4 eV.

***Ground electronic state rovibrational properties.*** The vibrational energy levels have been obtained using the following formula:


$$E\;=\;\sum_{i}\omega_{i}^{RCCSD(T)–F12}\;(v_{i}+\frac{1}{2})+\sum_{i\geq j}x_{ij}^{MP2}\;(v_{i}+\frac{1}{2})(v_{j}+\frac{1}{2})\;(2)$$

where ω _i_ represents the RCCSD(T)-F12 harmonic fundamentals and x _ij_ are the MP2 anharmonic constants. These last values were computed using VPT2 theory and harmonic force fields MP2/AVQZ (in the case of the small radicals) and MP2/AVTZ (in the case of CH _3_COCH _2_). In Table 5, the computed fundamentals are compared with previous measured values in Ar ^16– 18^ and p-H _2_^20^ matrices, and in the gas phase ^21– 28^.

**Table 5.** Anharmonic fundamentals ^a, b, c^ (in cm ^-1^).

CH _3_CO				
			Calc.	Obs. ^d^ [Ref]
ν( a’)	ν _1_	CH _3_ st	2988	2989.1 (19) ^20^
	ν _2_	CH _3_ st	2919	2915.6(29) ^20^
	ν _3_	CO st	**1900**	1880.5 (100) ^20^; 1875 ^17^; 1842 ^18^
	ν _4_	CH _3_ b	1427	1419.9(80) ^20^; 1420 ^17^
	ν _5_	CH _3_ b	1331	1323.2(109) ^20^; 1329 ^17, 18^
	ν _6_	HCC b	**1021**	
	ν _7_	CC st	833	836.6(45) ^20^
	ν _8_	OCCb	464	468.1(28) ^20^
ν( a”)	ν _9_	CH3 st	**2997**	2990.3(42) ^20^
	ν _10_	CH _3_ b	1429	1419.9(80) ^20^
	ν _11_	CH _3_ b	**931**	
	ν _12_	CH _3_ tor	82	
**CH _2_CHO**				
ν( a’)	ν _1_	CH st	3133	
	ν _2_	CH st	3035	
	ν _3_	CH st	**2813**	2827.91 ^21^
	ν _4_	CCO st	**1465**	1528 ^22– 26^
	ν _5_	CH _2_ b	**1435**	1486 ^25^
	ν _6_	OCH b	**1365**	24, 25
	ν _7_	CC st	1132	1143 ^22, 23, 25– 27^
	ν _8_	CC st	950	957 ^25^
	ν _9_	CCO b	495	22, 23, 25– 27
ν( a”)	ν _10_	H _4_ wag	954	703 ^25^
	ν _11_	CH _2_ wag	729	557 ^25^
	ν _12_	CH _2_ tor	402	402(4) ^25^
**CH _3_COCH _2_**				
ν( a’)	ν _1_	CH _2_ st	3145	
	ν _2_	CH _3_ st	2024	
	ν _3_	CH _2_ st	3040	
	ν _4_	CH _3_ st	**2927**	
	ν _5_	CO st	**1541**	1554.1 ^16^, 1558.9 ^16^
	ν _6_	CH _3_ b	**1449**	
	ν _7_	CH _2_ b	**1440**	1419.32 ^16^
	ν _8_	CH _3_ b	1365	1377.51 ^16^
	ν _9_	CCH b	1243	1247 ^28^
	ν _10_	CCH b	1051	
	ν _11_	CCH b	910	
	ν _12_	CC st	813	
	ν _13_	OCC b	521	515 ^28^
	ν _14_	CCCb	385	
ν( a”)	ν _15_	CH _3_ st	2971	
	ν _16_	CH _3_ b	**1440**	
	ν _17_	CH _3_ b	1001	
	ν _18_	CH _2_ wag	732	
	ν _19_	CH _3_ b	500	
	ν _20_	CH _2_ tor	343	
	ν _21_	CH _3_ tor	77	

a) st= stretching; b=bending;wag=wagging; tor=torsion. b) 16– 18 Measured in Ar matrix; 20 in pH _2_ solid; 21– 28 in the gas phase. c) Emphasized in bold transitions for which important Fermi displacements are predicted. d) Experimental uncertainties, when available, are given in parentheses in units of the last quoted digit.

The ground vibrational state rotational constants and the centrifugal distortion constants are shown in Table 6. The rotational constants were computed using the RCCSD(T)-F12 equilibrium parameters of Table 3 and the following equation, proposed and verified in previous studies ^42, 65– 67^:

**Table 6.** Vibrational ground state rotational constants (in MHz) and centrifugal distortion constants corresponding to the symmetrically reduced Hamiltonian parameters (III ^r^ representation ^a^).

	CH _3_CO		CH _2_CHO	
	Calc.	Exp. ^b^	Calc.	Exp. ^c^
A _0_	84094.27	82946.73	66773.15	66677.85679(159)
B _0_	9952.09	9955.46	11445.40	11447.0460(55)
C _0_	9427.41	9426.95	9762.15	9758.9065(53)
Δ _J_	0.009448	0.01188 (21)	0.009422	0.0096468(22)
Δ _K_	2.756343		1.312964	1.307
Δ _JK_	0.124574		-0.084559	-0.083045(137)
d _1_	-0.000948		-0.002006	0.0021215(101)
d _2_	0.000512		-0.000129	0.0384(26)
H _J_	0.000041		0.000014	
H _K_	0.897964		0.078700	
H _JK_	-0.001033		-0.000287	
H _KJ_	-0.630725		-0.006429	
h _1_	0.000004		0.000007	
h _2_	-0.000014		0.000001	
h _3_	0.000001		0.000000	
**CH _3_COCH _2_**				
A _0_	10949.35		H _J_	0.000024
B _0_	9054.05		H _K_	-0.000014
C _0_	5110.78		H _JK_	-0.000088
Δ _J_	0.010134		H _KJ_	0.000078
Δ _K_	0.004463		h _1_	0.000006
Δ _JK_	-0.013811		h _2_	0.000003
d _1_	-0.000763		h _3_	0.000002
d _2_	-0.000596			

a)      The z-axis was selected to coincide with the x-Eckart axis in CH _3_CO and CH _2_CHO, and with the z-Eckart axis in CH _3_COCH _2_. b)      Computed from the A, (B±C)/2, and D values of Ref. 14. c)      Ref 15.


$$\begin{array}{l} {\text{B}}_{\text{0}}\;\text{=}\;{\text{B}}_{\text{e}}\;\text{(RCCSD(T)-F12/AVTZ)}\;\text{+}\;{\text{ΔB}}_{\text{e}}^{\text{core}}\;\text{(RCCSD(T)/CVTZ)}\\ \;\text{+}\;{\text{ΔB}}_{\text{vib}}\;\text{(MP2/AVnZ)} \end{array}\;(3)$$

Here, ∆B _e_^core^ takes into account the core-valence-electron correlation effect on the equilibrium parameters. It can be evaluated as the difference between B _e_(CV) (calculated by correlating both core and valence electrons) and B _e_(V) (calculated by only correlating the valence electrons). ∆B _vib_ represents the vibrational contribution to the rotational constants derived from the VPT2 α _ir_ vibration-rotation interaction parameters.

Rotational parameters of CH _3_CO were compared with the experimental values of Hirota *et al.* ^14^, whereas those of CH _2_CHO were compared with the data of Endo *et al.* ^15^. For this second radical, the agreement between computed and measured rotational constants is excellent (A _0_^CAL^-A _0_^EXP^ = 95.3 MHz, B _0_^CAL^-B _0_^EXP^ = -1.7 MHz, and C _0_^CAL^-C _0_^EXP^ = 3.2 MHz). However, for CH _3_CO, the concurrence is also excellent for B _0_ and C _0_, but the A _0_ result is outside tolerance limits (A _0_^CAL^-A _0_^EXP^ = 1147.5 MHz, B _0_^CAL^-B _0_^EXP^ = -3.4 MHz, and C _0_^CAL^-C _0_^EXP^ = 0.5 MHz). Generally, for many molecules, the computed B _0_ and C _0_ using Equation (3) are more accurate than A _0_. However, the difference expressed as A _0_^CAL^-A _0_^EXP^ = 1147.5 MHz is too large in comparison to what was expected. Since the three rotational constants were computed simultaneously, the error could be derived from the experiments and from the effective Hamiltonian used for assignments. For methyl isocyanate ^68^, we found a similar situation, where it is proven that previous experimental works provided very contradictory A _0_ constants.

### The far infrared region

For the three radicals, the energy levels corresponding to the large amplitude motions were computed using a variational procedure of reduced dimensionality, where the vibrational coordinates responsible for the non-rigidity were considered to be separable from the remaining vibrations. Then, an adiabatic approximation is applied on the basis of the vibrational energies. Since the method takes into consideration the minimum interconversion describing the tunneling effects in the barriers, it is more suitable for nonrigid species than VPT2, although this last theory provides a useful preliminary depiction. With VPT2, the methyl torsional fundamental of CH _3_CO was computed to be 82 cm ^-1^ and the fundamental frequency of the central bond torsion of CH _2_CHO was found at 402 cm ^-1^ (see Table 5). The CH _3_COCH _2_ radical presents two interacting internal rotations which VPT2 frequencies computed to be 77 cm ^-1^ (CH _3_ torsion) and 343 cm ^-1^ (CH _2_ torsion). In principle, models in one-dimension (1D) or two-dimensions (2D) seems sufficient.

However, as we employed a flexible model where the remaining vibrational modes were allowed to be relaxed during the torsions, a third vibrational mode, the CH _2_ wagging, must be considered explicitly as it is strongly coupled with the CH _2_ torsion. Fermi interactions, predicted using VPT2, show that the separability between the CH _2_ wagging and the CH _2_ torsion is not suitable. Then, CH _2_CHO and CH _3_COCH _2_ require (at least) to use 2D and a three-dimension (3D) model, respectively. For the most general case, the 3D Hamiltonian for J=0 must be defined as ^57– 59^:


$$\begin{array}{l} H(\theta_{1},\theta_{2},\alpha )\;=\;-\sum_{i=1}^{3}\sum_{j=1}^{3}\left(\frac{\partial }{\partial q_{i}}\right)\;B_{q_{i}q_{j}}\;(\theta_{1},\theta_{2},\alpha )\;\left(\frac{\partial }{\partial q_{j}}\right)\;+\;V^{eff}\;(\theta_{1},\theta_{2},\alpha )\\ \;{\text{q}}_{\text{i}}{\text{q}}_{\text{j}}{\text{=θ}}_{\text{1}}{\text{,θ}}_{\text{2}}\text{,α} \end{array}\;(4)$$

where B _qiqj_ (θ _1_,θ _2_, α) are the kinetic energy parameters ^57– 59^; the effective potential is the sum of three terms:


$${\text{V}}^{\text{eff}}\;{\text{(θ}}_{\text{1}}{\text{,θ}}_{\text{2}}\text{,α)}\;{\text{=V(θ}}_{\text{1}}{\text{,θ}}_{\text{2}}\text{,α)}\;\text{+}\;{\text{V}}^{\text{,}}{\text{(θ}}_{\text{1}}{\text{,θ}}_{\text{2}}\text{,α)}\;\text{+}\;{\text{V}}^{\text{ZPVE}}{\text{(θ}}_{\text{1}}{\text{,θ}}_{\text{2}}\text{,α)}\;(5)$$

Here, V(θ _1_,θ _2_, α) represents the *ab initio* potential energy surface; V’(θ _1_,θ _2_, α) is the Podolsky pseudopotencial and V ^ZPVE^(θ _1_,θ _2_, α) represents the zero point vibrational energy correction ^57– 59^. For the 1D and 2D model, the corresponding operators depending on θ _1_ (CH _3_CO) or θ _2_ and α (CH _2_CHO) can be easily derived from Equation (4) removing variables. A possible analytical expression for the potential can be the product of a double Fourier series and a Taylor series:


$$\begin{array}{l} {\text{V}}^{\text{eff}}{\text{(θ}}_{\text{1}}{\text{,θ}}_{\text{2}}\text{,α)=}\sum \sum \sum \;[{\text{A}}_{\text{mnl}}\;{\text{cos(3mθ}}_{\text{1}}\text{)}\;{\text{cos(2nθ}}_{\text{2}}\text{)}\;{\text{α}}^{\text{21}}\;\text{+}\;{\text{A}}_{\text{m-nl}}\;{\text{cos(3mθ}}_{\text{1}}\text{)}\;\text{sin}\;{\text{(2n+1)θ}}_{\text{2}}\;{\text{α}}^{\text{21+1}}\\ \;\text{+}\;{\text{A}}_{\text{-m-nl}}\;{\text{sin(3mθ}}_{\text{1}}\text{)}\;{\text{sin(2nθ}}_{\text{2}}\text{)}\;{\text{α}}^{\text{21}}\;\text{+}\;{\text{A}}_{\text{-mnl}}\;{\text{sin(3mθ}}_{\text{1}}\text{)}\;{\text{cos(2n+1)θ}}_{\text{2}}\;{\text{α}}^{\text{21+1}}]\\ \;\text{m=0,1,2;}\;\text{n=0,1,2;}\;\text{l=0,1,2,3,4} \end{array}\;(6)$$

Similar expressions can be used for the kinetic parameters. In the most general 3D case, the Hamiltonian symmetry properties correspond to the totally symmetric representation of G _12_^69^, the molecular symmetry group (MSG) of the CH _3_COCH _2_ radical. Three-dimensional series corresponding to the six representations, four non degenerate A _1_^’^, A _1_”, A _2_’ and A _2_”, and two double-degenerate, E’ and E” are presented in Table 7. The MSG of the radical CH _3_CO is G _6_ with three irreducible representations, and that of CH _2_CHO is G _4_ with four irreducible representations A ^+^, A ^-^, B ^+^ and B ^-^ (based on effects of the symmetry operators (56) and E*).

**Table 7.** Three-dimensional wavefunctions according to MSG G _12_ (m,n,l=0,1,2,..).

A _1_’	E _a_’
cos(3mθ _1_) cos(2nθ _2_) α ^2l^ cos(3mθ _1_) sin (2n+1)θ _2_ α ^2l+1^	cos(3m±1)θ _1_ cos(2nθ _2_) α ^2l^ cos(3m±1)θ _1_ sin (2n+1)θ _2_ α ^2l+1^
sin(3mθ _1_) sin(2nθ _2_) α ^2l^ sin(3mθ _1_) cos(2n+1)θ _2_ α ^2l+1^	cos(3m±1)θ _1_ sin(2nθ _2_) α ^2l^ cos(3m±1)θ _1_ cos(2n+1)θ _2_ α ^2l+1^
**A _2_’**	**E _b_’**
cos(3mθ _1_) sin(2nθ _2_) α ^2l^ cos(3mθ _1_) cos(2n+1)θ _2_ α ^2l+1^	sin(3m±1)θ _1_ sin(2nθ _2_) α ^2l^ sin(3m±1)θ _1_ cos(2n+1)θ _2_ α ^2l+1^
sin(3mθ _1_) cos(2nθ _2_) α ^2l^ sin (3mθ _1_) sin(2n+1)θ _2_ α ^2l+1^	sin(3m±1)θ _1_ cos(2nθ _2_) α ^2l^ sin (3m±1)θ _1_ sin(2n+1)θ _2_ α ^2l+1^
**A _1_”**	**E _a_”**
cos(3mθ _1_) sin(2nθ _2_) α ^2l+1^ cos(3mθ _1_) cos(2n+1)θ _2_ α ^2l^	cos(3m±1)θ _1_ sin(2nθ _2_) α ^2l+1^ cos(3m±1)θ _1_ cos(2n+1)θ _2_ α ^2l^
sin(3mθ _1_) cos(2nθ _2_) α ^2l+1^ sin(3mθ _1_) sin(2n+1)θ _2_ α ^2l^	cos(3m±1)θ _1_ cos(2nθ _2_) α ^2l+1^ cos(3m±1)θ _1_ sin(2n+1)θ _2_ α ^2l^
**A _2_”**	**E _b_”**
cos(3mθ _1_) cos(2nθ _2_) α ^2l+1^ cos(3mθ _1_) sin(2n+1)θ _2_ α ^2l^	sin(3m±1)θ _1_ cos(2nθ _2_) α ^2l+1^ sin(3m±1)θ _1_ sin(2n+1)θ _2_ α ^2l^
sin(3mθ _1_) sin(2nθ _2_) α ^2l+1^ sin(3mθ _1_) cos(2n+1)θ _2_ α ^2l^	sin(3m±1)θ _1_ sin(2nθ _2_) α ^2l+1^ sin(3m±1)θ _1_ cos(2n+1)θ _2_ α ^2l^

 The kinetic parameters and the *ab initio* potential energy surface were determined from a grid of N selected geometries, corresponding to selected values of the independent coordinates. When 1D, 2D, or 3D models are employed, 1, 2 or 3 internal coordinates are frozen at selected values, whereas 3N _a_-7 (1D), 3N _a_-8 (2D) and 3N _a_-9 (3D) (N _a_=number of atoms) are allowed to be relaxed in all the calculated N geometries. For the CH _3_COCH _2 _radical:

a)  Geometries corresponding to four values of the H5C2C1C3 dihedral angle (0°, 90, -90° and 180°) were selected. The methyl torsional coordinate is defined as:

                             θ _1_=(H5C2C1C3+ H6C2C1C3+ H7C2C1C3-360º)/3

For CH _3_CO, the angles H _x_C2C1O3 (x=4,5,6) define the methyl torsional coordinate.

**Figure 3.**
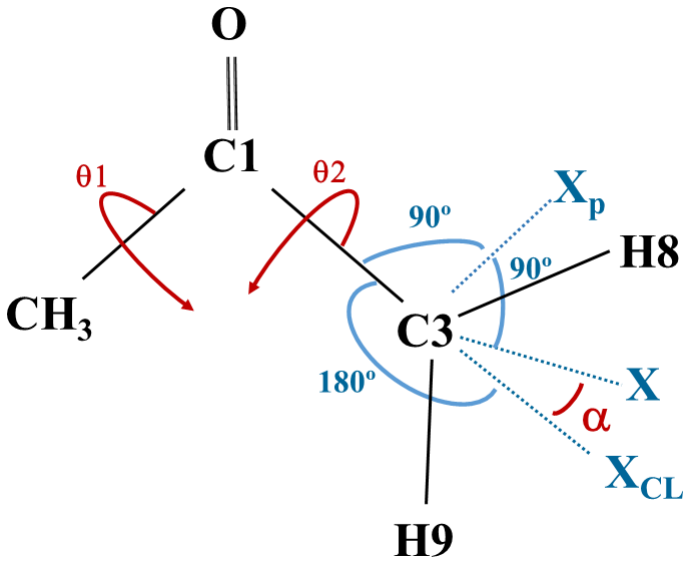
The ghost atoms defining the CH _2_ torsional (X _p_C3C1C2) and the wagging (XC3X _CL_) coordinates, in CH _3_COCH _2_.

b)  The procedure for defining the coordinates involving the CH _2_ group comprises 3 ghost atoms, X _p_, X _CL_, and X. X _p_ and X defining, respectively, the torsional and wagging coordinates assuring the optimization of 3N _a_-8 in CH _2_CHO and 3N _a_-9 in CH _3_COCH _2_ using the available GAUSSIAN ^47^ and MOLPRO software ^52^. Figure 3 shows the distributions of real and ghost atoms in CH _3_COCH _2_.

During the geometry optimization, X _p_ and X _CL_ were frozen in the plane defined by the three carbon atoms; C3-X could wag outside the plane formed by C3C1C2 but remained perpendicular to C3-X _p_; the C3-X _p_ and C3-X _CL_ “bonds” stood perpendicular and collinear to C3-C1, respectively; the real atoms H8 and H9 remained in a plane defined by the X, C3, and X _p_ atoms. Then, the independent α and θ _2 _variables and the corresponding selected values were defined as:


$$\begin{array}{l} {\text{α=<XC3X}}_{\text{CL}}\;\text{(α=}0^{\circ },\;\pm 15^{\circ },\;\pm 30^{\circ },\;\pm 45^{\circ }\;)\\ {\text{θ}}_{\text{2}}\text{=}\;{\text{X}}_{\text{p}}\;\text{C3C1C2}\;{\text{(θ}}_{\text{2}}=180^{\circ },150^{\circ },120^{\circ },90^{\circ }) \end{array}$$

A similar procedure was used for CH _2_CHO.

**Figure 4.**
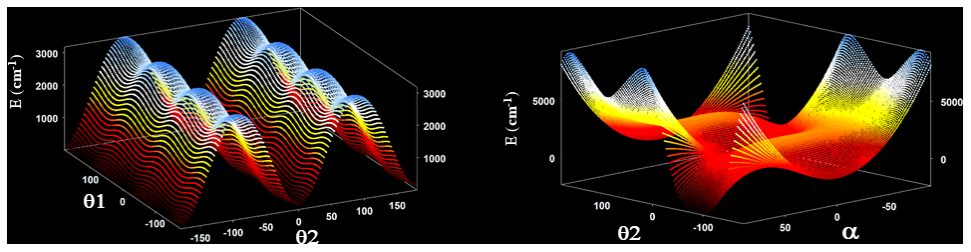
Two dimensional cuts of the 3D potential energy surface of CH _3_COCH _2_.

 The linear fit of the N *ab initio* energies to Equation (6) (R ^2^=0.99999; σ=1.096 cm ^-1^ (CH _3_COCH _2_)) produced V(θ _1_,θ _2_,α). The kinetic parameters B _q1q2_(θ _1_,θ _2_,α) and the pseudopotential V‘(θ _1_,θ _2_, α) were determined using the procedure described in 57 and 58 for the N geometries and fitted to an expansion formally identical to Equation (6). To determine V ^ZPVE^(θ _1_,θ _2_,α), harmonic frequencies were computed in all the N geometries. Details are shown in 56. The expansion coefficients of the effective potential are provided in Table 8. The A _000_ coefficients of the kinetic parameters are shown in Table 9. Figure 4 displays two bidimensional cuts of the 3D-effective potential of CH _3_COCH _2_. On the left side of the figure, the two coordinates correspond to the CH _3_ and CH _2_ torsions. On the right side, the two coordinates are those involving the CH _2_ group (torsion and wagging).

**Table 8.** Expansion coefficients of the potential energy surfaces (in cm ^-1^) ^a^.

CH _3_CO											
A _l_			M	A _l_		M		A _l_		M	
66.538			0	-71.867		3		5.329		6	
**CH _2_COH**											
**A _nl_**		**N**	**L**	**A _nl_**		**N**	**L**	**A _nl_**		**N**	**L**
2308.050		0	0	0.572		2	2	-4.161		-1	1
-1958.054		2	0	-0.6x10 ^-4^		2	4	0.7x10 ^-4^		-1	3
-55.340		4	0	-0.046		4	2	-3.838		-3	1
38.721		6	0	0.1x10 ^-4^		4	4	-0.11x10 ^-3^		-3	3
0.589		0	2	0.001		6	2	0.534		-5	1
0.10x10 ^-3^		0	4	-0.2x10 ^-5^		6	4	-0.1x10 ^-4^		-5	3
**CH _2_COCH _3_**											
**A _mnl_**	**N**	**M**	**L**	**A _mnl_**	**N**	**M**	**L**	**A _lmn_**	**N**	**M**	**L**
1936.238	0	0	0	-0.12x10 ^-3^	2	0	4	0.1x10 ^-5^	4	3	4
-1432.135	2	0	0	0.069	4	0	2	0.008	-1	3	1
-61.140	4	0	0	-0.4x10 ^-4^	4	0	4	-0.1x10 ^-4^	-1	3	3
-2.543	6	0	0	0.019	6	0	2	-1.108	-3	3	1
-149.629	0	3	0	-0.10x10 ^-4^	6	0	4	-0.5x10 ^-4^	-3	3	3
-0.521	0	6	0	0.062	-1	0	1	-0.033	-5	3	1
0.379	0	0	2	0.007	-1	0	3	0.5x10 ^-4^	-5	3	3
0.23x10 ^-3^	0	0	4	2.883	-3	0	1	0.038	-2	-3	2
72.044	2	3	0	0.001	-3	0	3	-0.1x10 ^-4^	-2	-3	4
-1.843	2	6	0	-0.112	-5	0	1	-0.013	-4	-3	2
-3.507	4	3	0	-0.5x10 ^-4^	-5	0	3	0.2x10 ^-5^	-4	-3	4
-0.023	4	6	0	-0.005	0	3	2	-0.056	-6	-3	2
0.378	6	3	0	0.1x10 ^-4^	0	3	4	0.3x10 ^-4^	-6	-3	4
0.347	6	6	0	-0.001	0	6	2	0.304	1	-3	1
-73.592	-2	-3	0	-0.023	2	3	2	0.3x10 ^-4^	1	-3	3
6.280	-4	-3	0	0.1x10 ^-5^	0	6	4	-1.382	3	-3	1
0.559	2	0	2	0.003	4	3	2	0.6x10 ^-3^	3	-3	3

a) M=3m; N=2n or 2n+1; L=2l or 2l+1; M ≥ 0 ⇒ *cos* 3mθ _1 ;_ M < 0 ⇒ *sin* 3mθ _1_; N ≥ 0 ⇒ *cos* Nθ _2 ;_ N < 0 ⇒ *sin* Nθ _2 ;_

**Table 9.** A _mnl_ (m=0, n=0, l=0) coefficients of the kinetic energy parameters (in cm ^-1^) ^a^.

	A _000_(B _aa_)	A _000_(B _bb_)	A _000_(B _cc_)	A _000_(B _ab_)	A _000_(B _ac_)	A _000_(B _bc_)
**CH _3_CO**	9.8515	-	-	-	-	-
**CH _2_CHO**		11.9647	35.8187	-	-	0.000
**CH _3_COCH _2_**	5.7225	9.9603	34.3821	-0.1792	0.000	0.000

a) a=CH _3_ torsion; b= CH _2_ torsion; c=CH _2_ wagging

The energy levels were computed variationally by solving the Hamiltonian of Equation (4), using the symmetry adapted series shown in Table 7 as trial wave functions. Details concerning the classification of the levels computed in 2D and 3D can be found in 11. Table 10 collects the variational energy levels and the transitions computed using VPT2.

**Table 10A.** Low-lying vibrational energy levels (in cm ^-1^) ^a^ of the CH _3_CO, CH _2_CHO and CH _3_COCH _2_ radicals computed variationally or using the vibrational second order perturbation theory. Variational energy levels are classified using the m, n and l quanta corresponding to the θ _1_, θ _2_, and α coordinates according to the excitation energy. The irreducible representations are given according to the MSG G _6_, G _4_ and G _12_, respectively.

CH _3_CO (G _6_)					CH _2_CHO (G _4_)					
m	Variational		VPT2		n l	Variational		VPT2		Exp. [Ref]
0	A _1_ E	0.0 2.997	ZPVE	-	0 0	A ^+^ B ^+^	0.000	ZPVE	-	
1	A _2_ E	104.265 76.656	ν _12_	82	1 0	A ^-^ B ^-^	398.436	ν _12_	402	402±4 ^33^
2	A _1_ E	132.180 185.760	2ν _12_	169				ν _9_	495	
3	A _2_ E	379.440 272.324	3ν _12_	261	0 1	A ^-^ B ^-^	745.727	ν _11_	729	557 ^33^
4	A _1_ E	379.849 507.146	4ν _12_	358	2 0	A ^+^ B ^+^	792.306	2ν _12_	782	
			ν _8_	464				ν _12_ν _9_	893	
			ν _12_ν _8_	549				ν _8_	950	
5	A _2_ E	821.806 654.566	5ν _12_	461				ν _10_	954	
			2ν _12_ν _8_	639				2ν _9_	993	
			3ν _12_ν _8_	734	1 1	A ^+^ B ^+^	1123.980	ν _12_ν _11_	1107	
6	A _1_ E	821.807 1008.819	6ν _12_	-				ν _7_	1132	
			ν _7_	833	3 0	A ^-^ B ^-^	1178.128	3ν _12_	1140	
			ν _11_	**931**				ν _11_ν _9_	1225	
			2ν _8_	929				ν _12_ν _8_	1348	
			ν _12_ν _7_	**915**				ν _12_ν _10_	1357	
			ν _12_ν _11_	**998**				ν _6_	**1365**	
						……….				
					0 2	A ^+^ B ^+^	1495.772	2ν _11_	**1459**	

a)      Emphasized in bold transitions where important Fermi displacements are predicted.

**Table 10B.** 

CH _3_COCH _2_ (G _12_)									
m n l	Variational		VPT2		m n l	Variational		VPT2	
0 0 0	A _1_’, A _1_” E’, E”	0.000 0.320	ZPVE	-				ν _21_ν _19_	579
1 0 0	A _2_’, A _2_” E’, E”	84.176 78.053	ν _21_	77				ν _21_ν _13_	597
2 0 0	A _1_’, A _1_” E’, E”	126.301 147.464	2ν _21_	146				3ν _21_ν _14_	622
3 0 0	A _2_’, A _2_” A _1_’, A _1_” E’, E” E’, E”	253.926 255.131 194.827 326.007	3ν _21_	207				2ν _21_ν _19_	651
0 1 0	A _2_’, A _2_” E’, E”	327.653 328.917	ν _20_	343				2ν _21_ν _13_	665
			ν _14_	385	0 2 0	A _1_’, A _1_” E’, E”	639.762 639.650	2ν _20_	683
1 1 0	A _1_’, A _1_” E’, E”	414.182 407.636	ν _21_ν _20_	410	0 0 1	A _2_’, A _2_” E’, E”	687.576 686.820	ν _18_	732
2 1 0	A _2_’, A _2_” E’, E”	459.559 412.652	2ν _21_ν _20_	468				ν _20_ν _14_	726
			ν _21_ν _14_	472				3ν _21_ν _19_	715
4 0 0	A _1_’, A _1_” A _2_’, A _2_” E’, E” E’, E”	507.358 507.467 479.993 527.420	4ν _21_	260				3ν _21_ν _13_	725
3 1 0	A _1_’, A _1_” A _2_’, A _2_” E’, E” E’, E”	586.289 587.815 613.918 660.732	3ν _21_ν _20_	519				2ν _20_ν _21_	739
			ν _19_	500		……….			
			2ν _21_ν _14_	551	0 0 2	A _1_’, A _1_”	1387.228	2ν _18_	1459
			ν _13_	521					

a) Emphasized in bold transitions where important Fermi displacements are predicted.

**Figure 5.**
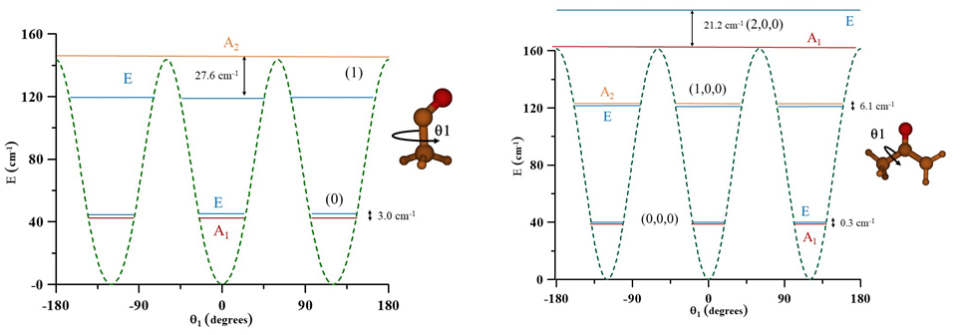
Methyl torsional energy levels of CH _3_CO and CH _3_COCH _2_.

 In CH _3_CO, the A _1_/E splitting of the ground vibrational state was evaluated to be 2.997 cm ^-1^, as was expected given the very low torsional barrier (V _3_=143.7 cm ^-1^). The methyl torsional fundamental ν _12_ (1←0), computed to be 82 cm ^-1^ with VPT2, present two components: 104.265 cm ^-1^ (A _2_←A _1_) and 73.659 cm ^-1^ (E←E). Figure 5 can help understand the distributions of levels and subcomponents. Excitation of the low amplitude motions ν _8_ (OCC bending), ν _7_ (C-C stretching), and ν _11_ (CH _3_ deformation) computed using VPT2 were inserted among the ν _12_ excitations, computed variationally.

In CH _2_CHO, the two independent fundamentals treated variationally were ν _12_ (1 0←0 0) and ν _11_ (0 1←0 0). Both were computed to be 402 cm ^-1^ and 729 cm ^-1^ using VPT2. The variational results were ν _12_ =398.436 cm-1 (A ^-^←A ^+^, B ^-^←B ^+^) and ν _11_ =745.727 cm ^-1^ (A ^-^←A ^+^, B ^-^←B ^+^). The first one was in agreement with the experimental data (402±4 cm ^-1^ ^35^) whereas large differences with the experimental work (557 cm ^-1^ ^35^) were observed for the CH _2_ wagging. Both works, experimental and theoretical, need to be revisited in the future to establish the wagging mode. The separation between splitting components (in the ground and first vibrational excited states) was very small due to the height of the torsional barrier V _2_=3838.7 cm ^-1^. For CH _2_CHO, the VPT2 model seems to be valid.

In CH _3_COCH _2_, the A/E CH _3_ torsional splitting of the ground vibrational state was computed to be 0.320 cm ^-1^. The two components of the three fundamentals ν _21_ (1 0 0 ←0 0 0), ν _20_ (0 1 0 ←0 0 0), and ν _18_ (0 0 1 ←0 0 0), were computed to be 84.176/77.733 cm ^-1^, 327.653/328.597 cm ^-1^, and 687.576/686.500 cm ^-1^, respectively.

## Conclusions

**Figure 6.**
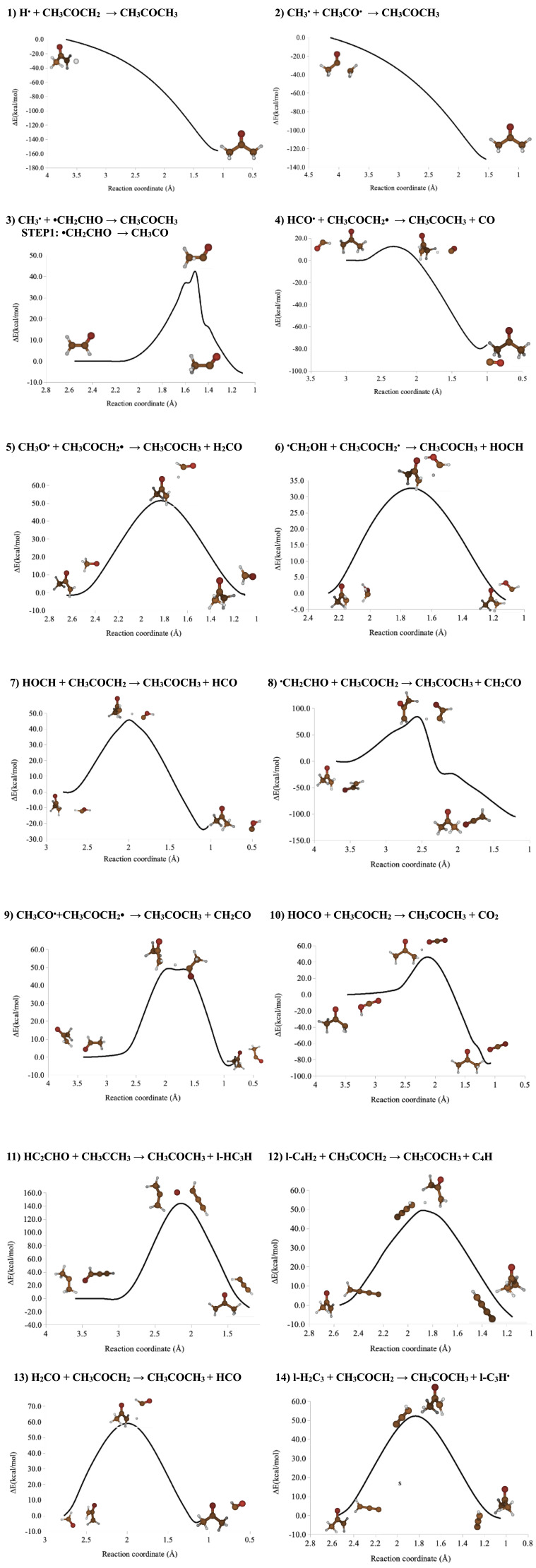
Minimum Energy Paths (MEP) corresponding to the formation processes of acetone computed at the M052X/6-31+G(d.p) level of theory.

 All the possible acetone formation routes starting from 58 selected reactants were automatically generated by the programme STAR. This analysis resulted in 75 exergonic processes involving CH _3_, CH _3_CO and CH _3_COCH _2_ radicals of which only 14 went through one or two steps and only two of them could be considered barrierless processes. The latter are the addition process H+ CH _3_COCH _2_ and CH _3_+CH _3_CO. Both showed similar kinetic rates. The 75 exergonic processes are collected in Table 11. Figure 6 shows the profiles of the fourteen simplest exergonic reactions processes.

**Table 11.** Exergonic reactions with reactants (∆G, in kcal/mol, at 10K and 0.1atm at CBS-QB3 level of theory; MNES is the maximum number of necessary elementary steps).

	Reactions	∆G	MNES
1	H ^•^ + CH _3_COCH _2_→ CH _3_COCH _3_	-94,9	1
2	CH _3_^•^ + CH _3_CO ^•^ → CH _3_COCH _3_	-83,4	1
3	CH _3_^•^ + ^•^CH _2_CHO → CH _3_COCH _3_	-89,2	2
4	HCO ^•^ + CH _3_COCH _2_→ CH _3_COCH _3_ +CO	-80,6	2
5	CH _3_O ^•^ + CH _3_COCH _2_→ CH _3_COCH _3_ +H _2_CO	-75,8	2
6	CH _2_OH ^•^ + CH _3_COCH _2_→ CH _3_COCH _3_ +H _2_CO	-67,3	2
7	CHOH + CH _3_COCH _2_ → CH _3_COCH _3_ + HCO ^•^	-60,3	2
8	^•^CH _2_CHO + CH _3_COCH _2_ → CH _3_COCH _3_ + CH _2_CO	-59,1	2
9	CH _3_CO ^•^ + CH _3_COCH _2_ → CH _3_COCH _3_ + CH _2_CO	-53,3	2
10	HOCO+ + CH _3_CCH _3_ → CH _3_COCH _3_ + HOC+	-32,3	2
11	HC _2_CHO + CH _3_CCH _3_ → CH _3_COCH _3_ + l-C _3_H _2_	-24,0	2
12	l-H _2_C _4_ + CH _3_COCH _2_→ CH _3_COCH _3_ +C _4_H ^•^	-8,9	2
13	H _2_CO+CH _3_COCH _2_→ CH _3_COCH _3_ +HCO ^•^	-7,4	2
14	l-C _3_H _2_ + CH _3_COCH _2_→ CH _3_COCH _3_ + l-C _3_H ^•^	-4,6	2
15	^3^CH _2_+CH _3_CHO→ CH _3_COCH _3_	-104,9	3
16	CH _2_CO+CH _3_CHO→ CH _3_COCH _3_ + CO	-27,2	4
17	CH _4_ +CH _2_CO→ CH _3_COCH _3_	-21,1	4
18	CH _3_O ^•^ + CH _3_CHOH → CH _3_COCH _3_ + H _2_O	-101,5	4
19	CH _2_OH ^•^ + CH _3_CHOH → CH _3_COCH _3_ + H _2_O	-92,9	4
20	CH _2_OH ^•^ + CH _3_CCH _3_ → CH _3_COCH _3_ + CH _3_^•^	-86,5	4
21	CH _2_CHOH + CH _3_CCH _3_ → CH _3_COCH _3_ + C _2_H _4_	-83,0	4
22	CH _3_^•^ + CH _3_CHOH → CH _3_COCH _3_ + H _2_	-75,3	4
23	CH _3_CHO + CH _3_CCH _3_ → CH _3_COCH _3_ + C _2_H _4_	-72,4	4
24	HOCO ^•^ + CH _3_CCH _3_ → CH _3_COCH _3_ + HCO ^•^	-71,2	4
25	HOCO+ + CH _3_CCH _3_ → CH _3_COCH _3_ + HCO+	-70,0	4
26	HCOOH + CH _3_CCH _3_ → CH _3_COCH _3_ + H _2_CO	-61,5	4
27	CHOH + CH _3_CCH _3_ → CH _3_COCH _3_ + ^3^CH _2_	-57,2	4
28	CH _3_COOH + CH _3_CCH _3_ → CH _3_COCH _3_ + CH _2_CHOH	-51,1	4
29	CH _3_O ^•^ + CH _3_CHCH _2_ → CH _3_COCH _3_ + CH _3_^•^	-27,4	4
30	C _6_H ^•^ + CH _3_CHOH → CH _3_COCH _3_ + C _5_	-26,5	4
31	CH _3_OH+ CH _3_CHO → CH _3_COCH _3_ + H _2_O	-21,7	4
32	OH ^•^ + CH _3_CHCH _2_ → CH _3_COCH _3_ + H ^•^	-14,8	4
33	CH _3_O ^•^ + CH _3_CHO→ CH _3_COCH _3_ + OH ^•^	-7,7	4
34	CH _3_CHO + ^•^CH _2_CHO → CH _3_COCH _3_ + HCO ^•^	-5,7	4
35	CH _3_CHO + CH _3_CHCH _2_ → CH _3_COCH _3_ + C _2_H _4_	-4,7	4
36	CH _3_CHO + CH _3_CHOH → CH _3_COCH _3_ + CH _2_OH ^•^	-2,8	4
37	CH _3_CCH + CH _3_CHO→ CH _3_COCH _3_ + C _2_H _2_	-1,8	4
38	CH ^•^ + CH _3_CHOH → CH _3_COCH _3_	-179,6	5
39	CH _3_OH + CH _3_CCH _3_ → CH _3_COCH _3_ + CH _4_	-94,9	5
40	HOCO ^•^ + CH _3_COCH _2_ → CH _3_COCH _3_ + CO _2_	-94,6	2
41	^3^CH _2_ + CH _3_CHOH → CH _3_COCH _3_ + H ^•^	-80,8	5
42	H _2_O + CH _3_CCH→ CH _3_COCH _3_	-37,8	5
43	l-C _3_H _2_ + CH _3_CHOH → CH _3_COCH _3_ + C _2_H ^•^	-35,9	5
44	C _4_H ^•^ + CH _3_CHOH → CH _3_COCH _3_ + C _3_	-33,8	5
45	H _2_C _6_ + CH _3_CHOH → CH _3_COCH _3_ + C5H ^•^	-32,7	5
46	CH _2_CO+CH _3_COOH→ CH _3_COCH _3_ +CO _2_	-31,7	5
47	C _2_H _2_+CH _3_COOH→ CH _3_COCH _3_ +CO	-30,7	5
48	CH _3_OH + CH _3_CHCH _2_ → CH _3_COCH _3_ + CH _4_	-27,3	5
49	l-H _2_C _4_+CH _3_COOH→ CH _3_COCH _3_ + C _3_O	-24,8	5
50	CH _2_OH• + CH _3_CHCH _2_ → CH _3_COCH _3_ + CH _3_^•^	-18,8	5
51	CH _2_CHOH + CH _3_CO ^•^ → CH _3_COCH _3_ + HCO ^•^	-10,5	5
52	^3^CH _2_ + CH _3_CH _2_OH→ CH _3_COCH _3_ + H _2_	-91,4	6
53	C _2_H _4_ + CHOH → CH _3_COCH _3_	-89,9	6
54	CH ^•^ + CH _3_CH _2_OH→ CH _3_COCH _3_ +H•	-85,7	6
55	CHOH + CH _3_CHOH → CH _3_COCH _3_ + OH ^•^	-55,6	6
56	l-H _2_C _4_ + CH _3_CHOH → CH _3_COCH _3_ + l-C _3_H ^•^	-22,6	6
57	CH _2_OHCHO + CH _3_CO ^•^ → CH _3_COCH _3_ + OHCO ^•^	-17,4	6
58	CH _2_CO + CH _3_CHOH → CH _3_COCH _3_ + HCO ^•^	-17,4	6
59	C _2_H _4_ + CH _3_CHOH → CH _3_COCH _3_ + CH _3_^•^	-16,9	6
60	CH _2_CHOH + CH _3_CHCH _2_ → CH _3_COCH _3_ + C _2_H _4_	-15,3	6
61	l-C _3_H ^•^ + CH _3_CHOH → CH _3_COCH _3_ + C _2_	-14,7	6
62	CH _2_CHOH + CH _3_CHOH → CH _3_COCH _3_ + CH _2_OH ^•^	-13,4	6
63	CH _2_OHCHO + CH _3_CHCH _2_ → CH _3_COCH _3_ + CH _2_CHOH	-10,4	6
64	CH _3_CCH + CH _3_COOH→ CH _3_COCH _3_ + CH _2_CO	-5,3	6
65	H _2_CO + CH _3_CHOH → CH _3_COCH _3_ + OH ^•^	-2,7	6
66	HOCO+ + CH _3_CHCH _2_ → CH _3_COCH _3_ + HCO ^+^	-2,4	6
67	H _2_CO+C _2_H _4_→ CH _3_COCH _3_	-36,8	7
68	CH _3_CH _2_OH + CHCO → CH _3_COCH _3_ + HCO ^•^	-28,5	7
69	CH _3_O ^•^ + C _2_H _4_→ CH _3_COCH _3_ + H ^•^	-17,7	7
70	CH _2_CHOH + CH _3_CHOH → CH _3_COCH _3_ + CH _3_O ^•^	-4,8	7
71	H _2_O + CH _3_CHCH _2_ → CH _3_COCH _3_ + H _2_	-1,3	7
72	C _2_H _2_+CH _3_OH→ CH _3_COCH _3_	-57,8	8
73	CH _2_OHCHO + CH _3_CHCH _2_ → CH _3_COCH _3_ + CH _3_CHO	-21,0	8
74	C _2_H _4_+CH _3_OH→ CH _3_COCH _3_ + H _2_	-18,2	8
75	CH _2_OH ^•^ + C _2_H _4_→ CH _3_COCH _3_ + H ^•^	-9,2	8

The geometrical and spectroscopic properties of some radicals involved in the processes, i.e. CH _3_CO, its isomer CH _2_CHO, and CH _3_COCH _2_, were determined by combining the two procedures and various levels of electronic structure theory. The first order properties such as geometries, equilibrium rotational constants, and harmonic fundamentals were determined using RCCSD(T). Using VPT2 and three MP2 anharmonic force fields, centrifugal distortion constants and anharmonic corrections for the spectroscopic parameters were computed.

As the three radicals are nonrigid species, the far infrared region was explored using a variational procedure depending on 1, 2, and 3 independent coordinates. Potential energy surfaces were computed at the RCCSD(T) levels of theory and were vibrationally corrected using MP2. This procedure takes into consideration the interconversion of the minima and allows to determine torsional splittings. In CH _3_CO, the A _1_/E splitting of the ground vibrational state, was evaluated to be 2.997 cm ^-1^, as was expected given the very low torsional barrier (V _3_ **=**143.7 cm ^-1^ **).**The methyl torsional fundamental ν _12_ (1←0) computed to be 82 cm ^-1^ with VPT2, presented two components to be 104.265 cm ^-1^ (A _2_←A _1_) and 73.659 cm ^-1^ (E←E). In CH _2_CHO, the two independent fundamentals ν _12_ (1 0←0 0) and ν _11_ (0 1←0 0) were computed to be ν _12_ =398.436 cm ^-1^ (A ^-^←A ^+^, B ^-^←B ^+^) and ν _11_ =745.727 cm ^-1^ (A ^-^←A ^+^, B ^-^←B ^+^). The separation between splitting components is very small due to the height of the torsional barrier V _2_=3838.7 cm ^-1^. For CH _2_CHO, the VPT2 model seems to be valid. In CH _3_COCH _2_, the A/E CH _3_ torsional splitting of the ground vibrational state was computed to be 0.320 cm ^-1^. The two components of the three fundamentals ν _21_ (1 0 0 ←0 0 0), ν _20_ (0 1 0 ←0 0 0), and ν _18_ (0 0 1 ←0 0 0), were computed to be 84.176/77.733 cm ^-1^, 327.653/328.597 cm ^-1^, and 687.576/686.500 cm ^-1^, respectively.

## Data availability

### Underlying data

All data underlying the results are included within the manuscript and no additional data are required.
