# Supplementary material for: Theoretical spectroscopic study of acetyl (CH 3CO), vinoxy (CH 2CHO), and 1-methylvinoxy (CH 3COCH 2) radicals. Barrierless formation processes of acetone in the gas phase
Source: Open Res Eur. 2022 Mar 18;1:116. Originally published 2021 Sep 30. [Version 2] doi: 10.12688/openreseurope.14073.2 (PMC10445905; doi:10.12688/openreseurope.14073.2)
Supplement: Peer review history

# Peer review: Reviewer response for version 2

**DOI:** 10.21956/openreseurope.15691.r28871

**Published:** 2022-04-04

**Authors:** Ha Vinh Lam Nguyen, Isabelle Kleiner

**Affiliations:** 1 CNRS, LISA, University of Paris Est Creteil, Paris, France

All scientific concerns have been addressed. Some issues concerning the formatting still remain but we believe that they will be fixed once the manuscript is finalized.

Is the study design appropriate and does the work have academic merit?

Yes

Is the work clearly and accurately presented and does it cite the current literature?

Yes

If applicable, is the statistical analysis and its interpretation appropriate?

Not applicable

Are all the source data underlying the results available to ensure full reproducibility?

No source data required

Are the conclusions drawn adequately supported by the results?

Yes

Are sufficient details of methods and analysis provided to allow replication by others?

Yes

Reviewer Expertise:

Rotational spectroscopy, microwave spectroscopy, large amplitude motions, internal rotation

We confirm that we have read this submission and believe that we have an appropriate level of expertise to confirm that it is of an acceptable scientific standard.

# Response

**Published:** 2022-04-04

**Authors:** Maria Luisa Senent Diez

**Affiliations:** IEM-CSIC, Spain

We thank the referees for the detailed work they have done and that has allowed us to significantly improve our article.

# Peer review: Reviewer response for version 2

**DOI:** 10.21956/openreseurope.15691.r28872

**Published:** 2022-04-01

**Authors:** Nicola Tasinato

**Affiliations:** 1 SMART Laboratory, Scuola Normale Superiore of Pisa, Pisa, Italy

I thank the authors for the clarifications and for the replies to my comments. The paper appears improved and hence I recommend publication in its current form.

Is the study design appropriate and does the work have academic merit?

Yes

Is the work clearly and accurately presented and does it cite the current literature?

Yes

If applicable, is the statistical analysis and its interpretation appropriate?

Not applicable

Are all the source data underlying the results available to ensure full reproducibility?

Partly

Are the conclusions drawn adequately supported by the results?

Yes

Are sufficient details of methods and analysis provided to allow replication by others?

Partly

Reviewer Expertise:

Experimental and theoretical spectroscopy; computational chemistry applied to structural, spectroscopic properties and chemical reactivity.

I confirm that I have read this submission and believe that I have an appropriate level of expertise to confirm that it is of an acceptable scientific standard.

# Response

**Published:** 2022-04-04

**Authors:** Maria Luisa Senent Diez

**Affiliations:** IEM-CSIC, Spain

We thank the referee for the detailed work he has done and that has allowed us to significantly improve our article.

# Peer review: Reviewer response for version 1

**DOI:** 10.21956/openreseurope.15168.r28017

**Published:** 2021-12-20

**Authors:** Ha Vinh Lam Nguyen, Isabelle Kleiner

**Affiliations:** 1 CNRS, LISA, University of Paris Est Creteil, Paris, France

This manuscript presents a theoretical study of three radicals undergoing large amplitude motion(s) (LAM), either three-fold as in the acetyl radical, two-fold as in the vinoxy radical, or a combination of both as in the 1-methylvinoxy radical with kinetic inputs to figure out reaction pathways to produce acetone from those radicals. Spectroscopic and geometrical properties such as rovibrational parameters, anharmonic vibrational transitions, and excitations to the low-lying excited states are provided to help the experimental detection of those species using rovibrational spectroscopy.

 This is an excellent study of importance in the high resolution molecular spectroscopic community. Not only for quantum chemists, spectroscopic theoretical results are also of big help when trying to make preliminary assignments in the rotational and far-infrared spectra. It might also be helpful to other fields whose inputs rely on high resolution spectroscopic data, i.e. atmospheric chemistry or astrochemistry, to judge the quality and accuracy of the acquired data. The research is original, state-of-the-art, and well-performed. The manuscript is also well-written, and we have no objection to its publication, provided some revision is made, as described below. Here, we emphasize the point of view of experimentalists in the high resolution molecular spectroscopic community, using quantum chemistry results as input for spectral analysis, and of spectroscopists with expertise in modeling spectra with LAMs.

 Imagining that we are aiming at studying the rotational or rovibrational spectrum of one of the three subject radicals, the first thing we will search in the paper would be the values of the rotational constants of the ground vibrational state, the geometry of the radical to have an idea about the orientation of the molecular fragment(s) undergoing the LAM, the tunneling barrier(s), and the coupling parameters between the electron spin and the rotation of the whole radical. The most important criterion for us is their accuracy, since laboratory detection of unstable species is very challenging from the experimental point of view. Purchasing the substance commercially with a reasonable purity is in no way possible, and producing it from precursors often yields a mixture of many other species which complicates very much the spectral assignment. Not taking into account the risk that the desired radical may not be produced.

 Therefore, regarding the rotational constants, the method given in Eq. (3) gives quite a different quality for the *A* rotational constants of CH _3_CO and CH _2_CO. It was found in many benchmark calculations for small to medium-sized molecules that the *B* and *C* rotational constants are often very well-predicted when compared to the values derived experimentally (e.g. K.L.K. Lee, M. McCarthy, *J. Phys.* *Chem. A* **2020**, *124*, 898-910). However, since the *A* rotational constant influences very much the spectral feature which is decisive for the assignment, the prediction accuracy of this parameter is crucial. To our point of view as rotational spectroscopists, 95.3 MHz difference is rather “good” (considering the value of *A* is large) than “excellent”. A difference of 1147.5 MHz is more than “outside the tolerance limits” – it is way too large to help the experimental search in such molecular systems. So the question for future high resolution spectroscopy  of CH _3_COCH _2_ is how much the theoretical prediction can be trusted. Does an additional correction necessary? The authors stated on page 9 that the discrepancy between the experimental and theoretical *A* values “could be derived from the experiments and from the effective Hamiltonian used for assignments” and gave an additional example on methyl isocyanate. This explanation is not very convincing. In the Fourier Transform microwave experiment, Hirota *et al.* only observed *K* = 0 *a*-type transitions. It is not so much "the experiments and the effective Hamiltonian" that can explain the discrepancy, but rather the present range of *J* and *K* values available in the dataset obtained from an experiment where large radicals are hard to produce. To our knowledge, the Hirota paper is the only rotational study involving radicals with internal rotation.

 Stay at this point, in Table 6, theoretical vibrational ground state rotational constants (in MHz) and centrifugal distortion constants corresponding to the symmetrically reduced Hamiltonian parameters in III ^r^ representation are compared with experimental constants from Hirota *et al*. But it is not clear where the authors took the experimental values for those three constants from? In Table 2 of Hirota *et al.*, we found A [82756.97] and the footnote says "value in brackets are assumed", so they probably fixed the *A* value, not fit. Furthermore, Hirota *et al.* used the RAM method, not PAM whereas theoretical values are in PAM. Did the authors transform the RAM constants of Hirota *et al.* into PAM? For the centrifugal distortion constants, they seem to be in I ^r^ representation in Hirota *et al.*, not in III ^r^. Those points should be clarified in the paper.

 The calculated value of 2.997 cm ^-1^ (almost 90000 MHz) for the A/E splitting for CH _3_CO seems very large (page 14). Can the authors compare their theoretical splitting value with the experimental splitting value observed by Hirota *et al.*?

 Another point missing from the manuscript is that, as found in the paper of Hirota *et al.*, for radicals the orbital and/or spin angular momenta of the unpaired electrons are known to coupled among them and with nuclear spin angular momenta. The orbital and/or spin angular momenta interact with the overall rotational angular momentum to split the rotational spectra of these species into fine and hyperfine components. No information on the corresponding parameters is available to give a hint on the hyperfine structures appearing on the A and E states and how they affect the spectrum. If possible, such information should be added in the paper. If not, comments about the fact that this information cannot be added are necessary.

 The choice of different methods to calculate the rotational constants should be clarified. The minimum energy paths are computed with DFT and 6-311+G(d,p) basis set, but it is not clear which DFT method the authors meant. The thermochemical properties are predicted with CB3-QB3, then the authors switched to M05-2X/6-311+G(d,p). The reason for choosing what to use is explained very well for the geometry optimizations and we expect the same for the calculations on energy paths and thermochemical properties.

 Finally, one wonders if relative intensity calculation can be provided for fundamental or overtone vibrational modes. This information is very valuable for future experimental infrared spectroscopy.

 There are some issues concerning the formatting and representation of the manuscript, which are all minor but need to be fixed:

- Page 4: “The work of Yamaguchi et al. focused on the CH _2_CHO, and CH _3_COCH _2_ radicals.” One half-sentence with more details as for the previous sentence of the paragraph is needed. By the way, the comma must be deleted.
- Page 5: MP2/VQZ is given twice. What is associated with “respectively”?
- Equation 1: The letter K on the exponential term must be a small k.
- Figure 1 and 2 captions: The figure captions are very modest and need to be extended. In Figure 2 and 5, 1 and 2 after q should be subscripted. Increase the size of the letters in Figures 5 and 6 (we can barely read Figure 5 and cannot read at all Figure 6).
- The last part of Table 6 on page 12 is not correct. Calc. on the third column should be deleted and Exp. should be Calc.
- Overall, there is some formatting scheme which we would use uniquely throughout the manuscript (parameters such as rotational constants, s, T, mu, Q, E, alpha, theta, V and quantum numbers such as J in italic, insert a space before and after = sign, use a real minus sign and not a hyphen for minus). Table 3 caption: MHZ should be MHz.
- Page 12, line -5: i and j in Bqiqj should be subscripted in relation to q.
- Table 3: H5C2=-H6C2: It may be better to put H6C2 in parentheses. Similar comment for other positions.

Is the study design appropriate and does the work have academic merit?

Yes

Is the work clearly and accurately presented and does it cite the current literature?

Yes

If applicable, is the statistical analysis and its interpretation appropriate?

Not applicable

Are all the source data underlying the results available to ensure full reproducibility?

No source data required

Are the conclusions drawn adequately supported by the results?

Yes

Are sufficient details of methods and analysis provided to allow replication by others?

Yes

Reviewer Expertise:

Rotational spectroscopy, microwave spectroscopy, large amplitude motions, internal rotation

We confirm that we have read this submission and believe that we have an appropriate level of expertise to confirm that it is of an acceptable scientific standard, however we have significant reservations, as outlined above.

# Response

**Published:** 2022-02-10

**Authors:** Maria Luisa Senent Diez

**Affiliations:** IEM-CSIC, Spain

We thank the referee for the work he has made and the interest he has shown in the article. Their comments have allowed us to improve our article. They confirm the interest of some results. Also, we would like to emphasize that we have found very interesting the discussion about the rotational parameters. (Reviewer comments in italics)

***Comment:*** *Therefore, regarding the rotational constants, the method given in Eq. (3) gives quite a different quality for the A rotational constants of CH _3_CO and CH _2_CO. It was found in many benchmark calculations for small to medium-sized molecules that the B and C rotational constants are often very well-predicted when compared to the values derived experimentally (e.g. K.L.K. Lee, M. McCarthy, J. Phys. Chem. A 2020, 124, 898-910). However, since the A rotational constant influences very much the spectral feature which is decisive for the assignment, the prediction accuracy of this parameter is crucial. To our point of view as rotational spectroscopists, 95.3 MHz difference is rather “good” (considering the value of A is large) than “excellent”. A difference of 1147.5 MHz is more than “outside the tolerance limits” – it is way too large to help the experimental search in such molecular systems. So the question for future high resolution spectroscopy of CH _3_COCH _2_ is how much the theoretical prediction can be trusted. Does an additional correction necessary? The authors stated on page 9 that the discrepancy between the experimental and theoretical A values “could be derived from the experiments and from the effective Hamiltonian used for assignments” and gave an additional example on methyl isocyanate. This explanation is not very convincing. In the Fourier Transform microwave experiment, Hirota et al. only observed K = 0 a-type transitions. It is not so much "the experiments and the effective Hamiltonian" that can explain the discrepancy, but rather the present range of J and K values available in the dataset obtained from an experiment where large radicals are hard to produce. To our knowledge, the Hirota paper is the only rotational study involving radicals with internal rotation.*

**Reply:** For CH2COH, the sentence has been rewritten: “For CH _2_CHO, the agreement between computed and measured rotational constants of B _0_ and C _0_ was excellent and tolerable in the case of A _0_ (A _0_^CAL^-A _0_^EXP^ = 95.3 MHz, B _0_^CAL^-B _0_^EXP^ = -1.7 MHz, and C _0_^CAL^-C _0_^EXP^ = 3.2 MHz)” Lee and McCarthy's publication discusses stable species with an even number of electrons. These systems are easier to obtain than open shell. Our experience applying equation 3 to neutral stable molecules is that the differences between measured and calculated values are ~10 MHz (in A _0_) and 3-5 MHz (in B _0_ and C _0_). A0 usually shows a slightly lower quality. A difference of A _0_^CAL^-A _0_^EXP^ = 95.3 MHz, B _0_^CAL^-B _0_^EXP^ = -1.7 MHz, and C _0_^CAL^-C _0_^EXP^ = 3.2 MHz), in a radical, as is the case of CH2CHO, can be considered a good result. However, a difference of 1147.5 MHz is not permissible. If more than one constant has a large error, it could be attributed to the chosen axis system. In this case, the rotation of the molecular frame system about an axis (i.e. changing A and B) can solve the problem. But, in our case, the CH _3_CO results for both C _0_ and B _0_ are excellent (A _0_^CAL^-A _0_^EXP^ = 1147.5 MHz, B _0_^CAL^-B _0_^EXP^ = -3.4 MHz, and C _0_^CAL^-C _0_^EXP^ = 0.5 MHz). Using ab initio calculations, all three constants are obtained at once because what we are calculating are the geometries.  An additional rotation about an axis can spoil one of the good constants. Then, the problem is not the selection of the axis, If only one rotational is wrong, the problem can come from two causes: one possibility is an error in the assignments. Another possibility can be that the theoretical model used for determining the Ae-A _0_ difference neglects important contributions. Our result stands out a problem that needs a further study. In fact, one of the applications of the ab initio calculations is that can stand new undetected problems. It is not only used as a single way to determine parameters. In the paper of Hirota *et al* [14], the A constant has not been fitted. It has been “assumed”. Perhaps (we do not know), they have been used the RAM equilibrium constant Ae computed using RCCSD(T)/cc-pVTZ (this is the level employed in Ref.14). A sentence “The authors of Ref [14] stood out  that the A constant was “assumed” whereas B _0_ and C _0_ were fitted using the observed lines” has been added to the manuscript.

***Comment:*** *Stay at this point, in Table 6, theoretical vibrational ground state rotational constants (in MHz) and centrifugal distortion constants corresponding to the symmetrically reduced Hamiltonian parameters in III ^r^ representation are compared with experimental constants from Hirota et al. But it is not clear where the authors took the experimental values for those three constants from? In Table 2 of Hirota et al., we found A [82756.97] and the footnote says "value in brackets are assumed", so they probably fixed the A value, not fit. Furthermore, Hirota et al. used the RAM method, not PAM whereas theoretical values are in PAM. Did the authors transform the RAM constants of Hirota et al. into PAM? For the centrifugal distortion constants, they seem to be in I ^r^ representation in Hirota et al., not in III ^r^. Those points should be clarified in the paper.*

*The calculated value of 2.997 cm ^-1^ (almost 90000 MHz) for the A/E splitting for CH _3_CO seems very large (page 14). Can the authors compare their theoretical splitting value with the experimental splitting value observed by Hirota et al.?. Another point missing from the manuscript is that, as found in the paper of Hirota et al., for radicals the orbital and/or spin angular momenta of the unpaired electrons are known to couple among them and with nuclear spin angular momenta. The orbital and/or spin angular momenta interact with the overall rotational angular momentum to split the rotational spectra of these species into fine and hyperfine components. No information on the corresponding parameters is available to give a hint on the hyperfine structures appearing on the A and E states and how they affect the spectrum. If possible, such information should be added in the paper. If not, comments about the fact that this information cannot be added are necessary. The choice of different methods to calculate the rotational constants should be clarified.*

**Reply:** There is a new sentence in the section where we comment on Hirota *et al*’s work “The experimental work of CH _3_CO also details the hyperfine structure of the rotational spectrum ^14^” With respect to the problem of the splitting, since Ref. [14] details hyperfine structure, we prefer to compare the internal rotation barrier than the splitting (V3 was computed to be 143.7 cm-1 whereas the experimental value of Hirota et al. is 139.958 (18) cm-1. The energy levels computed from ab initio potential energy surfaces can have an error of 3 cm ^-1^, very accurate for the ab initio calculations. A sentence “The V _3 _ barrier of CH _3_CO can be compared with the experimental data of Hirota et al [14] (V _3_=139.958 (18) cm ^-1^)” has been added to the paper. The equation (3) employed for the determination of the rotational constants has been tested in many previous papers, some of them are in the list of references [Refs. 42, 67-68]. Be is computed with a very prestigious method RCCSD(T)-F12, the effect of the core electrons is added using RCCSD(T), with a core valence correlation consistent basis set, because we believe that the F12 correction is not relevant for internal shells. The less accurate part, the vibrational correction has been computed at low levels of theory to avoid a very large computational effort. Our computation of torsional levels does not consider the hyperfine structure because we do not compute the rotational structure. However, in Table 6 (now Table 7) the computed rotational parameters are given in the Principal Axes and compared to the experimental ones. The experimental rotational constants from the Rho -Axes Method have been transformed to the Principal Axes. We have eliminated the centrifugal constant of Ref.[14] shown in Table 6 (now Table 7) in the old manuscript. The comparison of the centrifugal parameters with the experimental ones is not reliable at all because only two experimental parameters were fitted as well as the A parameter was assumed. In addition, the experimental centrifugal constants are given in the A-reduced Hamiltonian and the calculated ones in the S-reduced Hamiltonian.

***Comment:*** *The minimum energy paths are computed with DFT and 6-311+G(d,p) basis set, but it is not clear which DFT method the authors meant. The thermochemical properties are predicted with CB3-QB3, then the authors switched to M05-2X/6-311+G(d,p). The reason for choosing what to use is explained very well for the geometry optimizations and we expect the same for the calculations on energy paths and thermochemical properties.*

**Reply:** We use very expensive methods for spectroscopy (RCCSD(T)-F12) and modest methods for reactivity. To evaluate the procedure to obtain rotational constants is possible to use our previous papers (in the references) were we obtain an accuracy of 5 MHz (in other molecules with more standard behaviours). The equilibrium geometries (for spectroscopy) were computed using RCCSD(T)-F12/AVTZ implemented in MOLPRO. Then the equilibrium rotational constants are computed at this level of theory which is very prestigious. The correction of the core is performed at the RCCSD(T) level of theory because we believe that here the F12 correction is not relevant. The vibrational correction has been evaluated using MP2/AVTZ  on the basis of the anharmonicities are less dependent on the correlation than the harmonic contributions. Whereas we tried to get very accurate spectroscopic properties, the aim of the performed description of the reactions was mainly qualitative. We agree with the referee that the kinetic parameters may be of low accuracy especially at very low temperatures. It has to be considered the number of processes studied to elucidate what are the more probable processes can be performed by comparing the different behaviours. The modest level of theory allows to distinguish between barrier and barrier-less processes. Some comments have been added to the text in the “computational methods” section.

***Comment:*** *Finally, one wonders if relative intensity calculation can be provided for fundamental or overtone vibrational modes. This information is very valuable for future experimental infrared spectroscopy.*

**Reply:** Intensities have been added to Table 5 which summarizes VPT2 results. We have not determined intensities using the variational wavefunction because, as we have not experimental data, we cannot assert about the quality of the energy levels. The variational computation of intensities is very hard and we only provide intensities if we are very confident on our frequencies. If laboratories show interest in our work, and they realize that these calculations help their work, we will provide more properties. In this case, we can do intensity calculations for the bands lying in concrete in some particular spectral regions.

***Comment:*** *There are some issues concerning the formatting and representation of the manuscript, which are all minor but need to be fixed: Page 4: “The work of Yamaguchi et al. focused on the CH _2_CHO, and CH _3_COCH _2_ radicals.” One half-sentence with more details as for the previous sentence of the paragraph is needed. By the way, the comma must be deleted.*  

**Reply:** New sentence: “The work of Yamaguchi *et al.* ^40^ focused on the excited electronic states of CH _2_CHO and CH _3_COCH _2_ radicals, was performed using multireference configuration interaction theory”.

***Comment:*** *Page 5: MP2/VQZ is given twice. 0What is associated with “respectively”?  Equation 1: The letter K on the exponential term must be a small k. Figure 1 and 2 captions: The figure captions are very modest and need to be extended. In Figure 2 and 5, 1 and 2 after q should be subscripted. Increase the size of the letters in Figures 5 and 6 (we can barely read Figure 5 and cannot read at all Figure 6). The last part of Table 6 on page 12 is not correct. Calc. on the third column should be deleted and Exp. should be Calc. Overall, there is some formatting scheme which we would use uniquely throughout the manuscript (parameters such as rotational constants, s, T, mu, Q, E, alpha, theta, V and quantum numbers such as J in italic, insert a space before and after = sign, use a real minus sign and not a hyphen for minus). Table 3 caption: MHZ should be MHz.  Page 12, line -5: i and j in Bqiqj should be subscripted in relation to q. Table 3: H5C2=-H6C2: It may be better to put H6C2 in parentheses. Similar comment for other positions.*  

**Reply:** Thank you very much for your help. Errors have been corrected.

# Peer review: Reviewer response for version 1

**DOI:** 10.21956/openreseurope.15168.r28019

**Published:** 2021-11-29

**Authors:** Nicola Tasinato

**Affiliations:** 1 SMART Laboratory, Scuola Normale Superiore of Pisa, Pisa, Italy

The paper reports a theoretical study on possible formation routes of acetone under astrochemical conditions, i.e. by considering barrierless reactions. A preliminary screening has been performed and finally attention has been put on two barrierless reactions involving one elementary step. For these, rate constants have been determined using VRC-VTST.

 Then, equilibrium geometries, rotational and centrifugal distortion constants have been computed for three radicals, CH _3_CO, CH _2_CO and CH _3_COCH _2_ by adopting CCSD(T)-F12 and MP2 levels of theory. For these radicals, anharmonic vibrational frequencies have been computed by using vibrational perturbation theory for the small amplitude motions (SAMs) and a variational procedure for large amplitude motions (LAMs), under the assumption that the latter ones can be decoupled from SAMs.

 The methodological procedure appears adequate for the spectroscopic characterization, even though the accuracy issue is not discussed. Since computed data can be used to drive/interpret experiments, it would be interesting to point out their accuracy. Furthermore, while some wavenumbers have been experimentally measured by using different techniques (from gas-phase vibronic spectra to matrix-isolation spectroscopy), no information is available concerning infrared intensities in the gas-phase. Given the lack of experimental data, this is an important gap to fill concerning the usability of data for astrochemical detections so I am wondering why authors did not provide intensities.

 Similarly, what is the estimated accuracy for the structural parameters reported in Table 3, and why dipole moment is evaluated at MRCI/CASSCF level while the remaining computations use CCSD(T) or MP2 theories? In this respect, I suggest also changing the title of Table 3 because it seems that the reported structural parameters are evaluated using MRCI/CASSCF. Please, also write explicitly the reference wavefunction used for CCSD(T) computations of the radicals.

 The methodology used for studying formation reactions can have some drawbacks that should be discussed in the text. While I do not enter on the reliability of the screening by the STAR software, I do not understand why geometries have been computed at CBS-QB3 level, that actually means B3LYP/6-31G(d) geometries, and energy profiles have been computed at M05-2X/6-311+G(d,p) level. Indeed, it has been pointed out that B3LYP geometries, especially when not augmented for dispersion interactions, cannot be reliable enough for energy evaluations, in particular for reaction complexes and transition states. In this respect, and considering the size of the investigated systems, a double-hybrid functional in conjunction with a triple-zeta basis set may appear a safer choice for reliable predictions. Furthermore, it has been demonstrated that even CBS-QB3 energies can present errors that, in turn, may cause unreliable kinetic parameters.

 Concerning the computation of rotational spectroscopic parameters, what are the levels of theory used for quartic and sextic centrifugal distortions? In comparing the computed rotational constants to experimental values, it is better referring to relative errors (%) because they are probably more useful for the purpose. Indeed, A is the largest rotational constants, and hence it usually gives the largest deviations in absolute terms, but this is not necessarily true when relative errors are considered. Hence, I do not agree with the consideration that “Generally, for many molecules the computed B _0_ and C _0_ are more accurate than A _0_”, and it may also be misleading for non-experts in the field. By the way, what are the tolerance limits referred to in the manuscript (“… but the A _0_ result is outside the tolerance limits”)? Rather, I’m wondering if the cause of the deviation (about 1.4%) is related to the presence of LAMs in the CH _3_CO. Indeed, LAMs can give rise to non-physical values of cubic and quartic force constants that, coupled to the fourth-order expansion of the potential energy are at the basis of the limits of VPT2 for floppy molecules. Cubic force constants are also used to evaluate the alpha-rovibrational corrections, in turn employed to obtain vibrational contributions to rotational constants. Hence, vibrational corrections can be biased by the presence of LAMs through unreliable cubic force constants. This also agrees with the fact that, the largest effects of LAMs are reported for the CH _3_CO radical.

 In the discussion of the variational computation of fundamental frequencies and splitting, again, what is the expected accuracy? I presume that three decimal digits represent a too optimistic accuracy so please reduce the number of significant digits accordingly (unit or one decimal at most are well suited probably) throughout all the manuscript. Furthermore, I suggest rephrasing the statement “Excitation of the low amplitude motions nu8… were inserted among the nu12 excitations computed variationally.” (page 14). If I understood right authors are referring to combination bands (e.g. nu8 + nu12) whose frequencies have been computed by mixing the variational outcome (for e.g. nu12) with the VPT2 result (for e.g. nu8). If this is the case, the x _8,12_ anharmonic constant is required for obtaining the nu8+nu12 frequency, but x _8,12_ may be biased as well by the presence of the nu12 LAM through the cubic and quartic force constants involving this normal mode, unless the force constants involving nu12 are removed. Could the authors describe in more detail the adopted procedure and, eventually, how the issue concerning anharmonic constants have been addressed? By the way, concerning this discussion, low amplitude motions should be small amplitude motions.

 On inspecting Table 10, a huge difference can be observed for the variational and VPT2 frequencies of the 4nu21 overtone of CH _3_COCH _2_. This difference appears not coherent with the remaining entries of the table, so I am wondering if this is true or there is a typo in the reported value. If real, please comment on the cause of this huge difference.

 Table 11 and Figure 6 should be placed and discussed in Section “Barrierless formation processes of acetone”. Furthermore, the different energy contributions to DeltaG should be detailed in Table 11 and, more importantly, activation energies should be reported. Also Figure 6 is not adequately described: some of the minimum energy paths show a very peculiar shape, in particular 3) and 8), and hence deserve to be commented.

 In Table 5, several references are given for the experimental values, but it is not clear the source of some values. For example, for CH2CHO the experimental frequency of nu4 is reported to be 1528 cm ^-1^ taken from refs. 22-26, but the value reported in ref. 22 is 1560 cm ^-1^. So it would be nice to have either a correspondence between the reported value and reference from which it is taken, or describe how the experimental value has been derived.

 Apart from the above main points, the manuscript is in general well written although some polishing may be required, and also the text should be carefully revised to fix typos. Just to cite few of them: on page 5, VQZ (line 1) and MP2/VQZ (line 5) are reported twice; in Table 5 the experimental frequencies of nu6 and nu9 of  CH _2_CHO are missing and only references are given; Table 11: use ‘.’ as decimal separator instead of ‘,’.

Is the study design appropriate and does the work have academic merit?

Yes

Is the work clearly and accurately presented and does it cite the current literature?

Yes

If applicable, is the statistical analysis and its interpretation appropriate?

Not applicable

Are all the source data underlying the results available to ensure full reproducibility?

Partly

Are the conclusions drawn adequately supported by the results?

Yes

Are sufficient details of methods and analysis provided to allow replication by others?

Partly

Reviewer Expertise:

Experimental and theoretical spectroscopy; computational chemistry applied to structural, spectroscopic properties and chemical reactivity.

I confirm that I have read this submission and believe that I have an appropriate level of expertise to confirm that it is of an acceptable scientific standard, however I have significant reservations, as outlined above.

# Response

**Published:** 2022-02-10

**Authors:** Maria Luisa Senent Diez

**Affiliations:** IEM-CSIC, Spain

We thank the referee for the work he has made and the interest he has shown in the article. His comments have allowed us to write a new improved version of the article. They confirm the interest of some of our results. (Reviewer comments in italics)

***Comment:*** *The methodological procedure appears adequate for the spectroscopic characterization, even though the accuracy issue is not discussed. Since computed data can be used to drive/interpret experiments, it would be interesting to point out their accuracy. Furthermore, while some wavenumbers have been experimentally measured by using different techniques (from gas-phase vibronic spectra to matrix-isolation spectroscopy), no information is available concerning infrared intensities in the gas-phase. Given the lack of experimental data, this is an important gap to fill concerning the usability of data for astrochemical detections so I am wondering why authors did not provide intensities.*

**Reply:** In the “computational section”, some comments have been added concerning the precision of the calculations corresponding to the different sections of the paper. As the referee comments, most of the calculations are predictions. As we cannot rely on the comparison with experimental data to clarify this point, we rely on the results obtained in previous studies of molecules with more experimental data, carried out with the same methodology. Intensities have been added to Table 5 (now Table 6) which summarizes VPT2 results. We have not determined intensities using the variational wavefunction because, we have no experimental data to assess the quality of the energy levels. The variational computation of intensities is very cumbersome and we only provide intensities if we are very confident on our frequencies.  If the laboratories show interest in our work, these calculations could help their work, we will provide more properties. In this case, we can calculate the intensities for the bands lying in a particular spectral region.

***Comment:*** *Similarly, what is the estimated accuracy for the structural parameters reported in Table 3, and why dipole moment is evaluated at MRCI/CASSCF level while the remaining computations use CCSD(T) or MP2 theories? In this respect, I suggest also changing the title of Table 3 because it seems that the reported structural parameters are evaluated using MRCI/CASSCF. Please, also write explicitly the reference wavefunction used for CCSD(T) computations of the radicals.*

**Reply:** Our experience in the computations of the dipole moment of many molecules (particularly, when the ground state has not a singlet multiplicity character) shows that multiconfigurational methods such as MRCI produce more accurate dipole moments than monoconfigurational methods such as MP2. Nevertheless, given the lack of experimental data, there is no way for a discussion about this point although we have chosen the more prestigious procedure.  Dipole moment calculations with CCSD follow more complex paths than MP2, however CCSD does not satisfy the Hellmann-Feynman theorem in the usual sense. The way is not the determination of expectation values. In addition, there is no certainty that the outcome with CCSD is better than the MRCI.  For the radicals, we are using RCCSD(T) methods implemented in Molpro. As we are using default options, we have not given more explanations.  The caption of Table 3 has been corrected.

***Comment:*** *The methodology used for studying formation reactions can have some drawbacks that should be discussed in the text. While I do not enter on the reliability of the screening by the STAR software, I do not understand why geometries have been computed at CBS-QB3 level, that actually means B3LYP/6-31G(d) geometries, and energy profiles have been computed at M05-2X/6-311+G(d,p) level. Indeed, it has been pointed out that B3LYP geometries, especially when not augmented for dispersion interactions, cannot be reliable enough for energy evaluations, in particular for reaction complexes and transition states. In this respect, and considering the size of the investigated systems, a double-hybrid functional in conjunction with a triple-zeta basis set may appear a safer choice for reliable predictions. Furthermore, it has been demonstrated that even CBS-QB3 energies can present errors that, in turn, may cause unreliable kinetic parameters.*

**Reply:** Whereas we tried to get very accurate spectroscopic properties, the aim of the performed description of the reactions was mainly qualitative. We agree with the referee that the kinetic parameters may be of low accuracy specially at very low temperatures. Nevertheless, we have considered a very large number of processes and we were able to elucidate the more probable reactions by comparing the thermodynamic parameters.  The modest level of theory allows to distinguish between barrier and barrier-less processes. Some comments have been added to the text in the “computational methods” section.

***Comment:*** *Concerning the computation of rotational spectroscopic parameters, what are the levels of theory used for quartic and sextic centrifugal distortions? In comparing the computed rotational constants to experimental values, it is better referring to relative errors (%) because they are probably more useful for the purpose. Indeed, A is the largest rotational constants, and hence it usually gives the largest deviations in absolute terms, but this is not necessarily true when relative errors are considered. Hence, I do not agree with the consideration that “Generally, for many molecules the computed B _0_ and C _0_ are more accurate than A _0_”, and it may also be misleading for non-experts in the field. By the way, what are the tolerance limits referred to in the manuscript (“… but the A _0_ result is outside the tolerance limits”)? Rather, I’m wondering if the cause of the deviation (about 1.4%) is related to the presence of LAMs in the CH _3_CO. Indeed, LAMs can give rise to non-physical values of cubic and quartic force constants that, coupled to the fourth-order expansion of the potential energy are at the basis of the limits of VPT2 for floppy molecules. Cubic force constants are also used to evaluate the alpha-rovibrational corrections, in turn employed to obtain vibrational contributions to rotational constants. Hence, vibrational corrections can be biased by the presence of LAMs through unreliable cubic force constants. This also agrees with the fact that the largest effects of LAMs are reported for the CH _3_CO radical. * 

**Reply:** The sentence “Generally, for many molecules the computed B _0_ and C _0_ are more accurate than A _0_” refers (only) to the formulae that we employ combining RCCSD(T)-F12 equilibrium constants, with MP2/AVTZ vibrational corrections, and RCCSD(T)/CVTZ corrections of the core-electrons.  Of course, this is not a general result because many authors follow other approximate procedures with other results. We have employed our method for many non-rigid molecules, and an “usual result” is that whereas disagreement with experiments are of few MHz (less than 6-7MHz) for B and C, for A, discrepancies are more relevant.  We refer for some papers where we have obtained an important agreement with experiments. The formulae has been verified in many previous studies such as those described in Refs.42, 67-68. Some of these studies were achieved in collaboration with laboratories. In addition, we refer to a paper where discrepancies for the A constant are very large, but B _0_ and C _0_ where obtained with very good accuracy. In this case, the comparison was performed using experimental data from 3 different groups who provide very different experimental A _0_. In particular, in the paper of Hirota *et al* [14], the A constant has not been fitted. It has been “assumed”. Perhaps (we do not know), they have used the RAM equilibrium constant Ae computed using RCCSD(T)/cc-pVTZ (this is the level employed in Ref.14). Of course the presence of LAMs and the limit of the cubic force field computed with MP2/AVTZ introduce discrepancies. A sentence “The authors of Ref [14] have indicated that the A constant has been “assumed” whereas B _0_ and C _0_ were fitted using the observed lines” has been added to the manuscript.

***Comment:*** *In the discussion of the variational computation of fundamental frequencies and splitting, again, what is the expected accuracy? I presume that three decimal digits represent a too optimistic accuracy so please reduce the number of significant digits accordingly (unit or one decimal at most are well suited probably) throughout all the manuscript. Furthermore, I suggest rephrasing the statement “Excitation of the low amplitude motions nu8… were inserted among the nu12 excitations computed variationally.” (page 14). If I understood right authors are referring to combination bands (e.g. nu8 + nu12) whose frequencies have been computed by mixing the variational outcome (for e.g. nu12) with the VPT2 result (for e.g. nu8). If this is the case, the x _8,12_ anharmonic constant is required for obtaining the nu8+nu12 frequency, but x _8,12_ may be biased as well by the presence of the nu12 LAM through the cubic and quartic force constants involving this normal mode, unless the force constants involving nu12 are removed. Could the authors describe in more detail the adopted procedure and, eventually, how the issue concerning anharmonic constants have been addressed? By the way, concerning this discussion, low amplitude motions should be small amplitude motions.*

**Reply:** Low instead small has been corrected   A sentence has been added: “By taking into consideration previous studies of closed-shell molecules performed with this model, we can indicate that the accuracy of the torsional fundamentals is of few wavenumber (errors < 5 cm ^-1^). The accuracy decreases with the energy and close to the tops of the barriers.  We provide 3 decimal digits because this allow distinguish the splittings.  To clarify the problem, we have rewritten the cited sentence “Excitation for transitions that cannot be computed using a three-dimensional model but are expected to lie in the studied spectral region, the VPT2 band center positions are given in Table 10. These corresponds to the small amplitude modes n _8_ (OCC bending), n _7_ (C-C stretching), and n _11_ (CH _3_ deformation).

***Comment:*** *On inspecting Table 10, a huge difference can be observed for the variational and VPT2 frequencies of the 4nu21 overtone of CH _3_COCH _2_. This difference appears not coherent with the remaining entries of the table, so I am wondering if this is true or there is a typo in the reported value. If real, please comment on the cause of this huge difference.*

**Reply:** It is not a typo, this is the obtained result but both theories that we are using have limitations. The 4nu21 was computed using the VPT2 anharmonic constants and v21 is a torsional mode. In addition, the radical has a very low barrier. Both numbers can carry out errors.

***Comment:*** *Table 11 and Figure 6 should be placed and discussed in Section “Barrierless formation processes of acetone”. Furthermore, the different energy contributions to DeltaG should be detailed in Table 11 and, more importantly, activation energies should be reported. Also Figure 6 is not adequately described: some of the minimum energy paths show a very peculiar shape, in particular 3) and 8), and hence deserve to be commented.*

**Reply:** As we explain before, our aim is to identify straightforward barrier-less processes for a very large number of reactions. Table 11 lists a total of 75 processes with DG <0 of the many that have been considered. To determine correct thermodynamic parameters for all of them or very well shaped figures is above our objectives. In addition, 73 involve several stages (MNES > 1), which makes them very unlikely, especially at low temperatures. We do not regard the activation energies because we look for barrier-less processes. Besides, Table 11 (now Table 2) and Fig. 6 (now Fig. 1) have been included in this section. So the other tables and figures have been changed accordingly to the new enumeration.

***Comment:*** *In Table 5, several references are given for the experimental values, but it is not clear the source of some values. For example, for CH2CHO the experimental frequency of nu4 is reported to be 1528 cm ^-1^ taken from refs. 22-26, but the value reported in ref. 22 is 1560 cm ^-1^. So it would be nice to have either a correspondence between the reported value and reference from which it is taken, or describe how the experimental value has been derived.*

**Reply:** The frequency of 1528 cm-1 has been measured in the gas phase using laser induce fluorescence. We collect (if it is possible) gas phase frequencies.

***Comment:*** *Apart from the above main points, the manuscript is in general well written although some polishing may be required, and also the text should be carefully revised to fix typos. Just to cite few of them: on page 5, VQZ (line 1) and MP2/VQZ (line 5) are reported twice; in Table 5 the experimental frequencies of nu6 and nu9 of CH _2_CHO are missing and only references are given; Table 11: use ‘.’ as decimal separator instead of ‘,’.*

**Reply:** Thank you very much for your help. Errors have been corrected.

***Comment:*** *Are sufficient details of methods and analysis provided to allow replication by others? Partly*

**Reply** *: *Some explanations have been added.

***Comment:*** *Are all the source data underlying the results available to ensure full reproducibility? Partly*

**Reply:** All the data required to assure reproducibility are available in the text with an exception that are the expansion coefficients of the torsional kinetic parameters. We provide the A _000_ coefficients which are the most significant. Remaining coefficients can alter very slightly the final frequencies.
